# Supplementary material for: A marine heatwave drives significant shifts in pelagic microbiology
Source: Commun Biol. 2024 Jan 24;7:125. doi: 10.1038/s42003-023-05702-4 (PMC10808424; doi:10.1038/s42003-023-05702-4)
Supplement: Supplementary file 2 — Supplementary Information [file 42003_2023_5702_MOESM2_ESM.pdf]

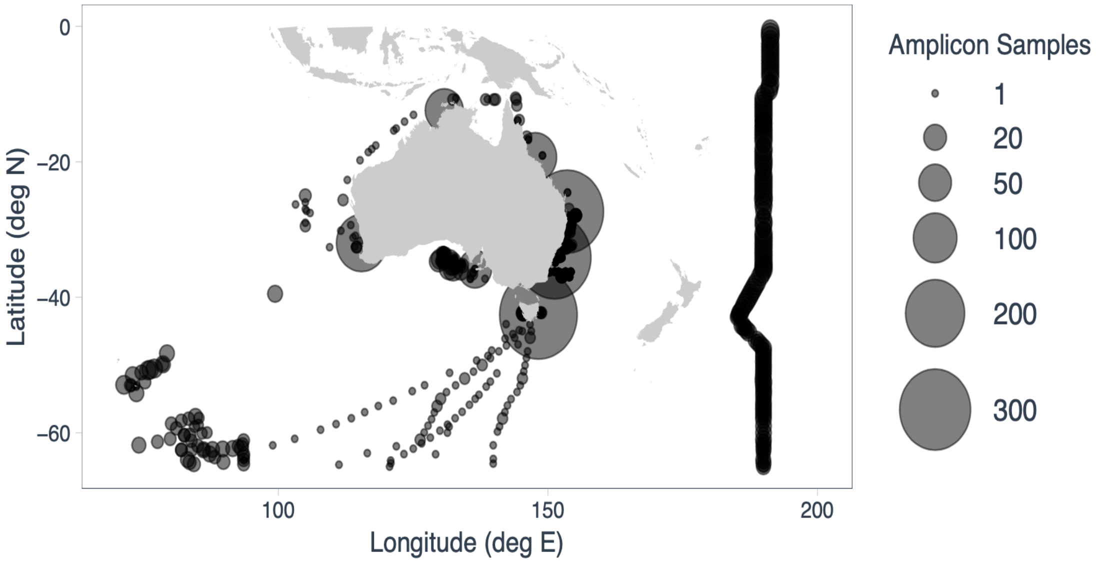

Supplementary Figure 1. Overview of the geographic distribution and number of samples from each location included in our dataset. Large circles are centred on Integrated Marine Observing System National Reference Stations.

a

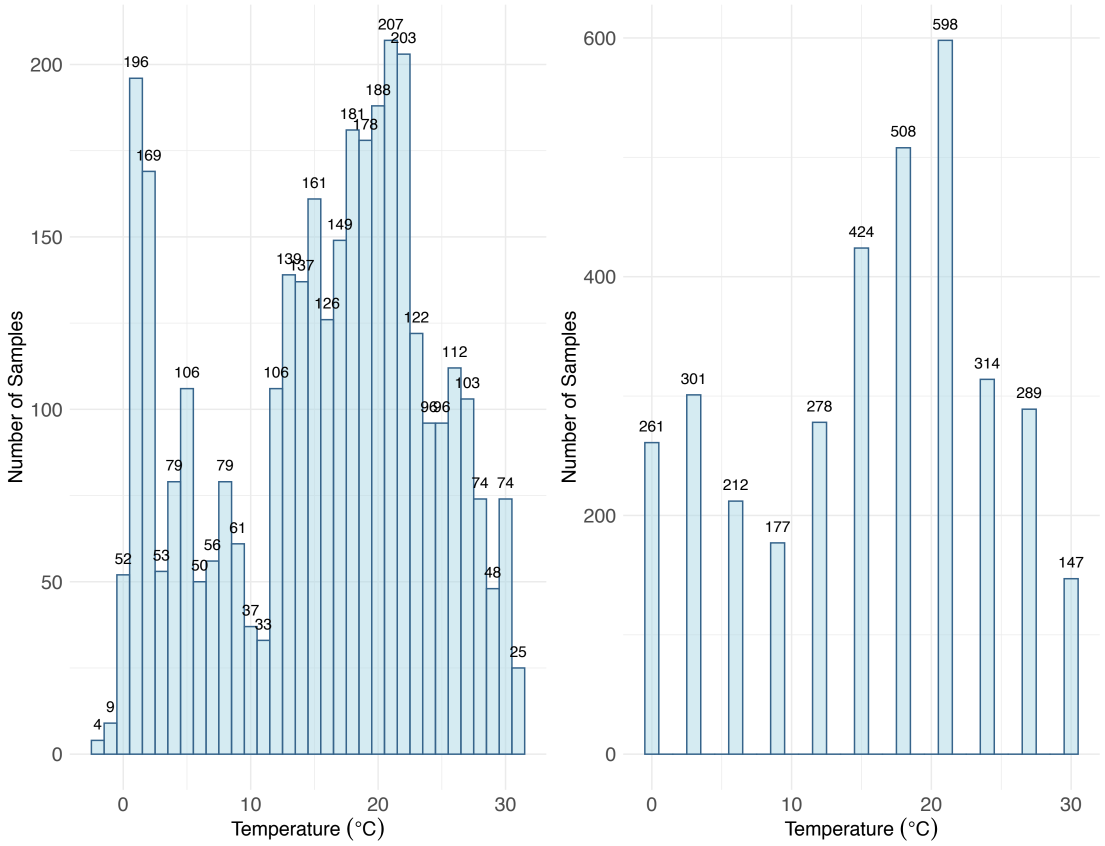

b

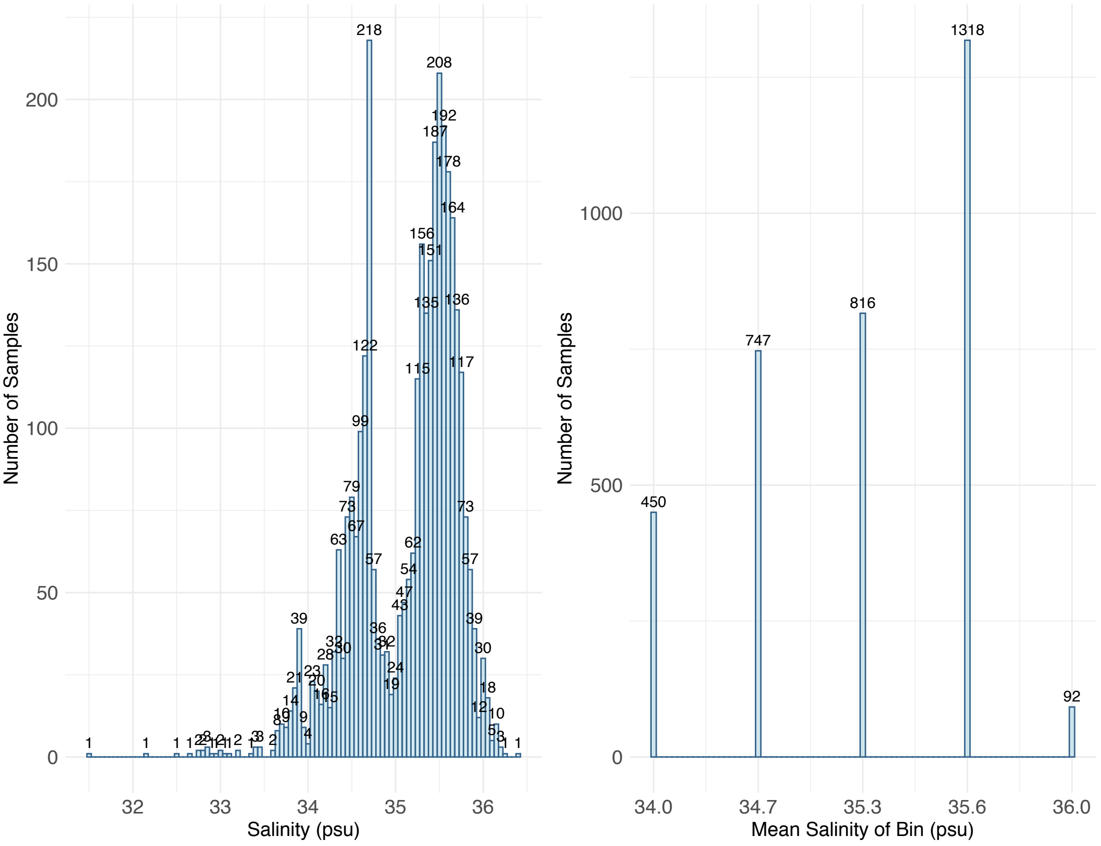
Number of Samples
Salinity (psu)
0
50
100
150
200
32
33
34
35
36
218
208
Number of Samples
Mean Salinity of Bin (psu)
0
500
1000
34.0
34.7
35.3
35.6
36.0
450
747
816
1318
92

c

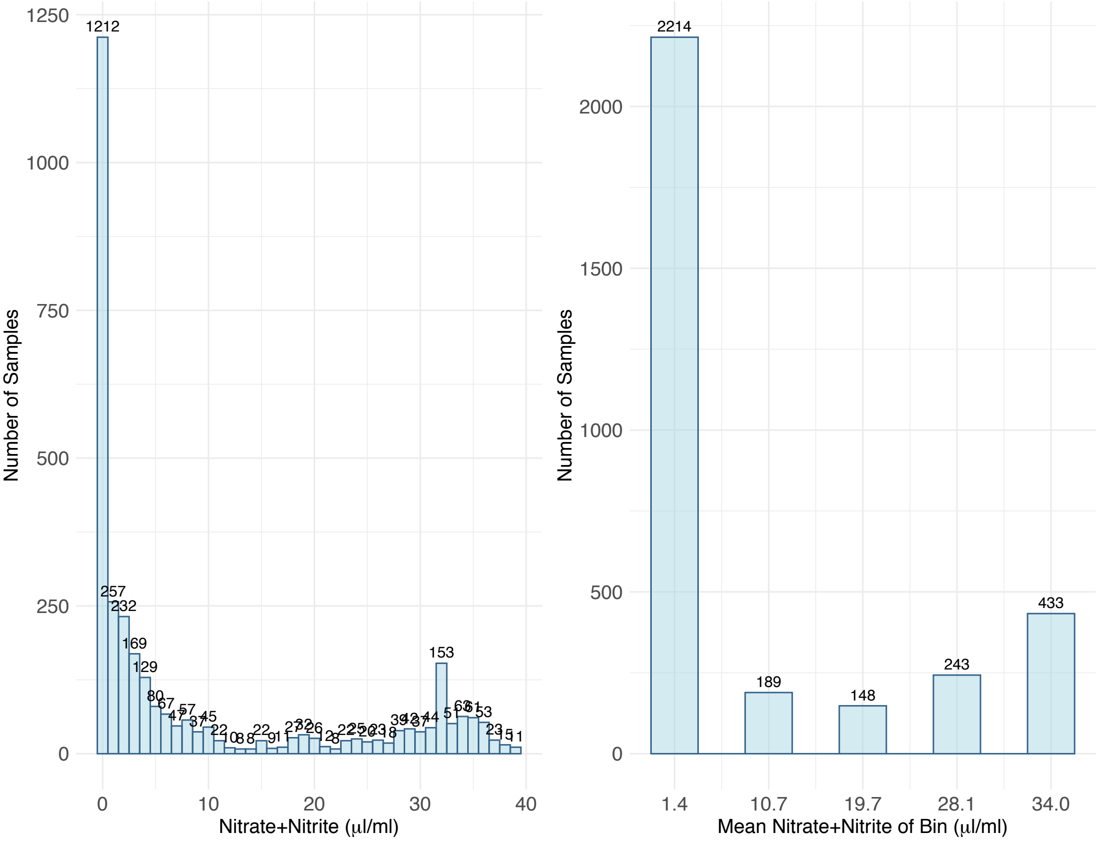
Number of Samples
Nitrate+Nitrite (μl/ml)
0
250
500
750
1000
1250
0
10
20
30
40
1212
257
232
169
129
153
Number of Samples
Mean Nitrate+Nitrite of Bin (μl/ml)
0
500
1000
1500
2000
1.4
10.7
19.7
28.1
34.0
2214
189
148
243
433

d

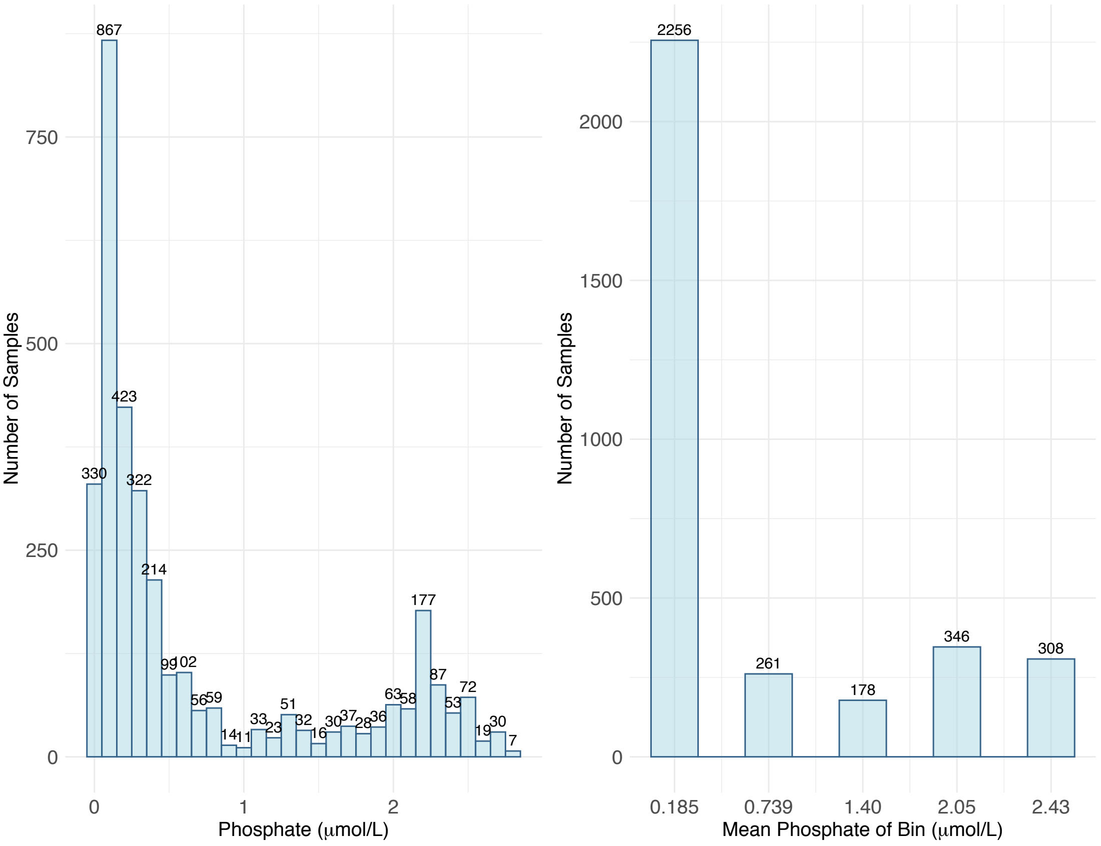

Number of Samples
Phosphate (μmol/L)
0
1
2
0
250
500
750
867
423
330
322
214
99
102
56
59
14
11
33
23
51
32
16
30
37
28
36
63
58
177
87
53
72
19
30
7
Number of Samples
Mean Phosphate of Bin (μmol/L)
0.185
0.739
1.40
2.05
2.43
0
500
1000
1500
2000
2256
261
178
346
308

e

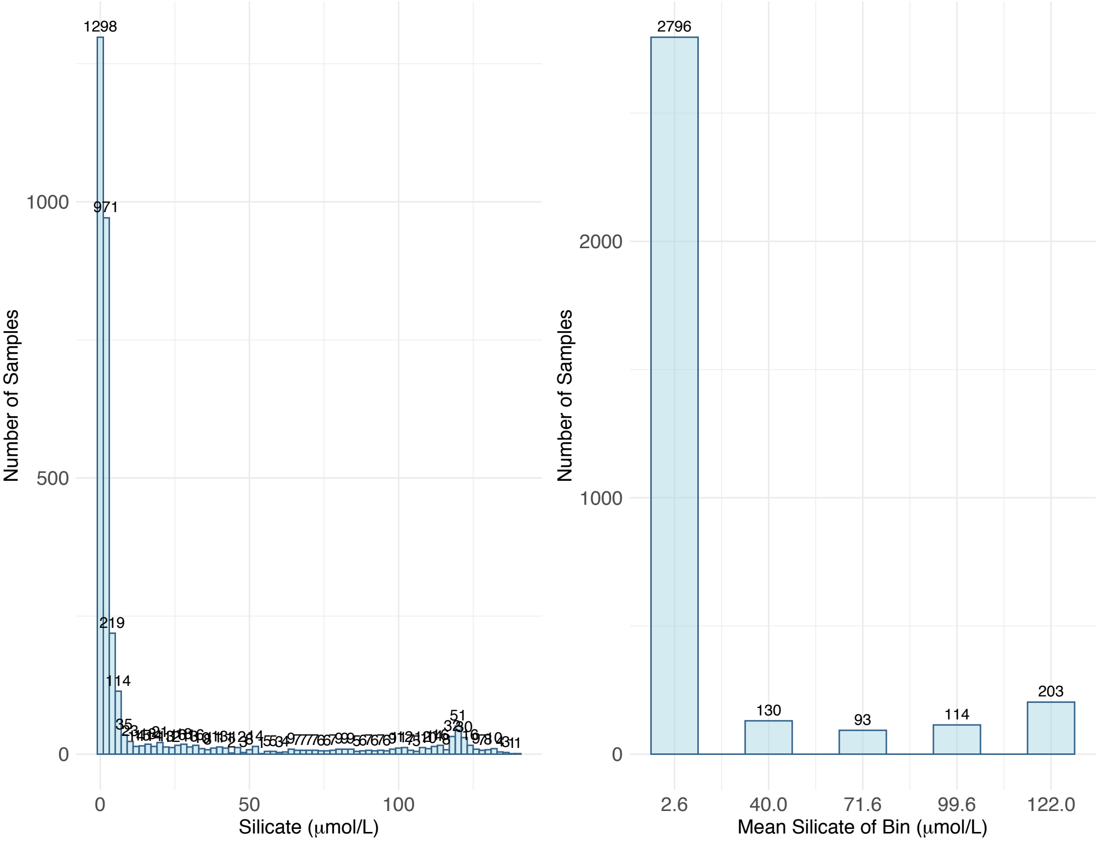

Number of Samples
Silicate (μmol/L)
0
50
100
0
500
1000
1298
971
219
114
35
51
Number of Samples
Mean Silicate of Bin (μmol/L)
2.6
40.0
71.6
99.6
122.0
0
1000
2000
2796
130
93
114
203

f

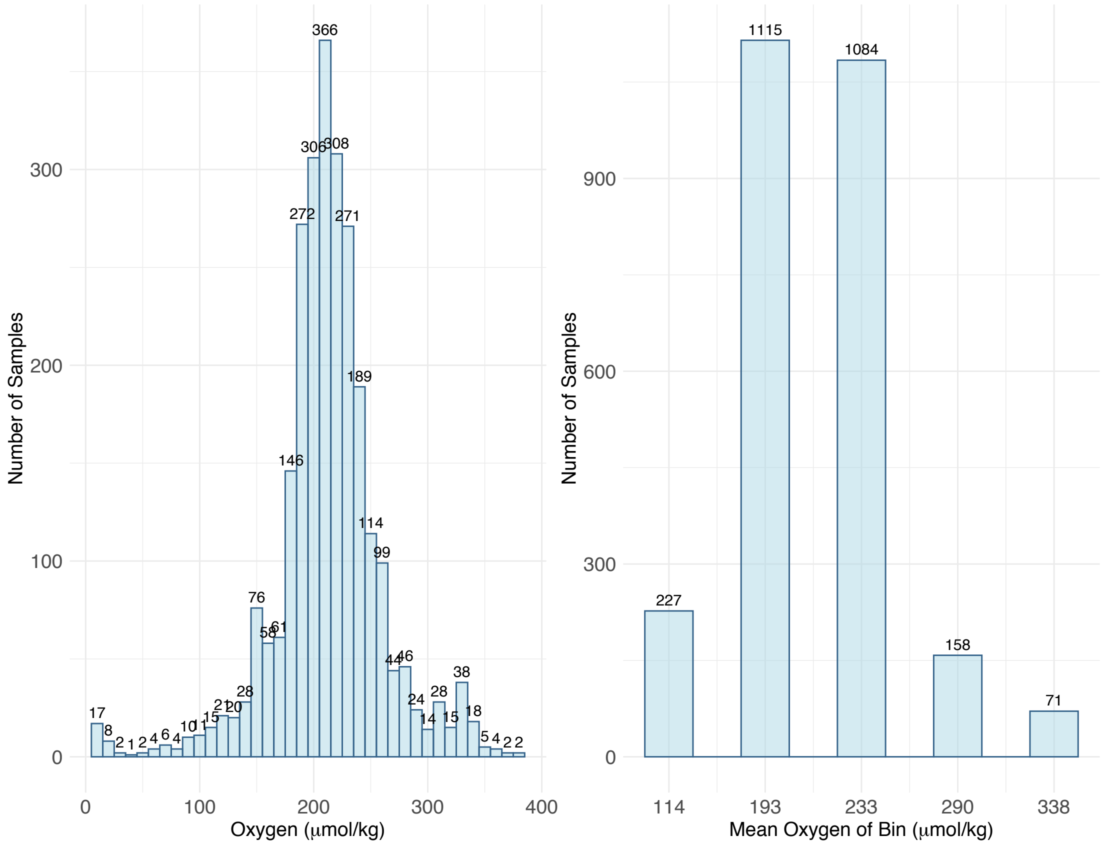

Supplementary Figure 2. Distribution of samples along environmental gradients and bins size and position used for 1000 x resampling during the generation of species level niche indices a) Temperature (n=3516), b) Salinity (n=3450), Cc) Nitrate+Nitrite (n=3367), d) Phosphate (n=3376), e) Silicate (n=3375), f) Oxygen (n=2675).

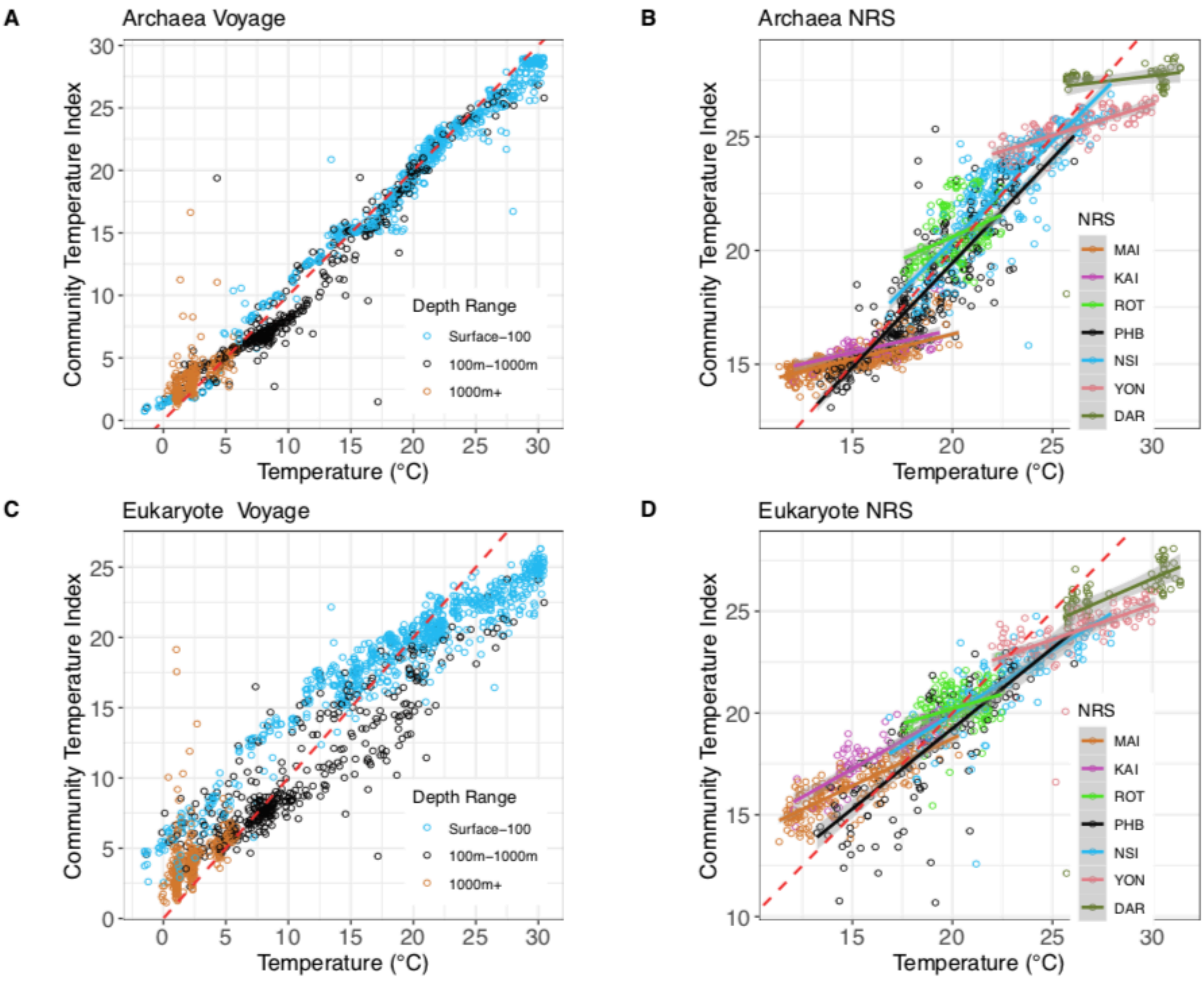

Supplementary Figure 3. Archaeal (A, n = 1468 and, B, n = 1607) and microbial eukaryote (C, n = 1492 and D, n = 1018) CTI against *in situ* environmental temperature of samples collected A,C) during oceanic voyages in the Southern Hemisphere and B,D) at IMOS National Reference Stations (NRS) time-series sites around the Australian continental shelf. Dashed red line represents a slope of one.

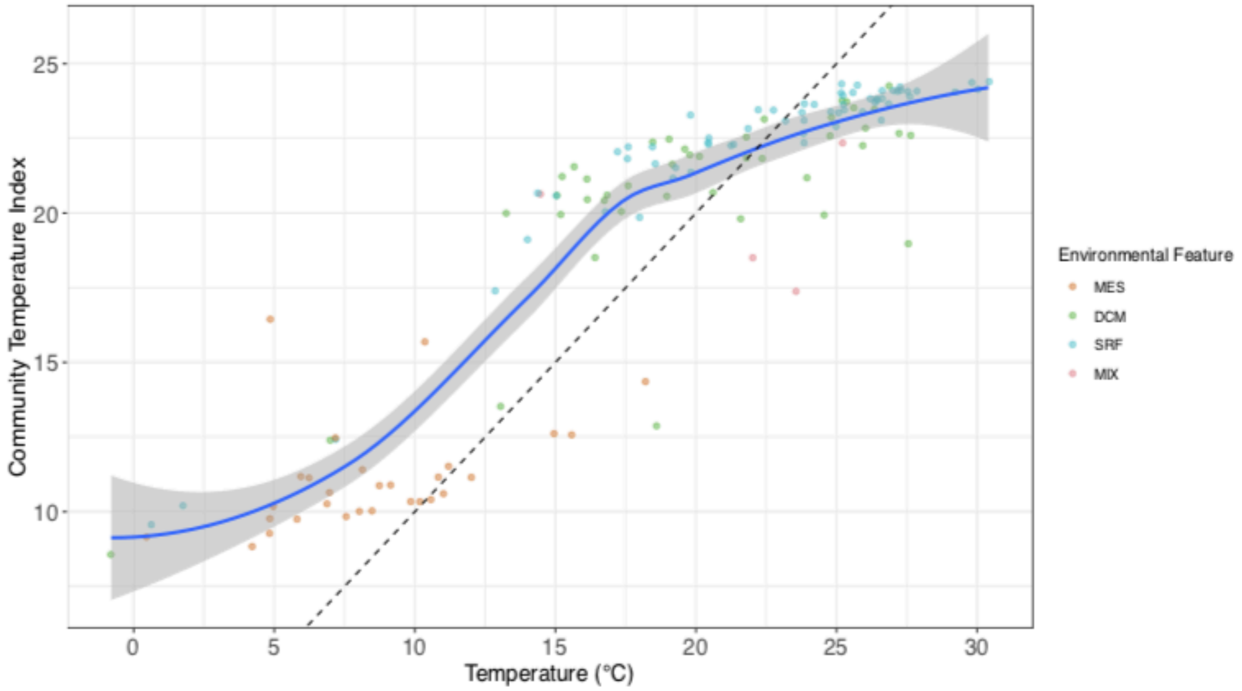

Supplementary Figure 4. The microbial CTI of samples (n=139) from the TARA Oceans expedition based on the mitag data and environmental variables obtained from ocean-microbiome.rmblr.de/companion.html.

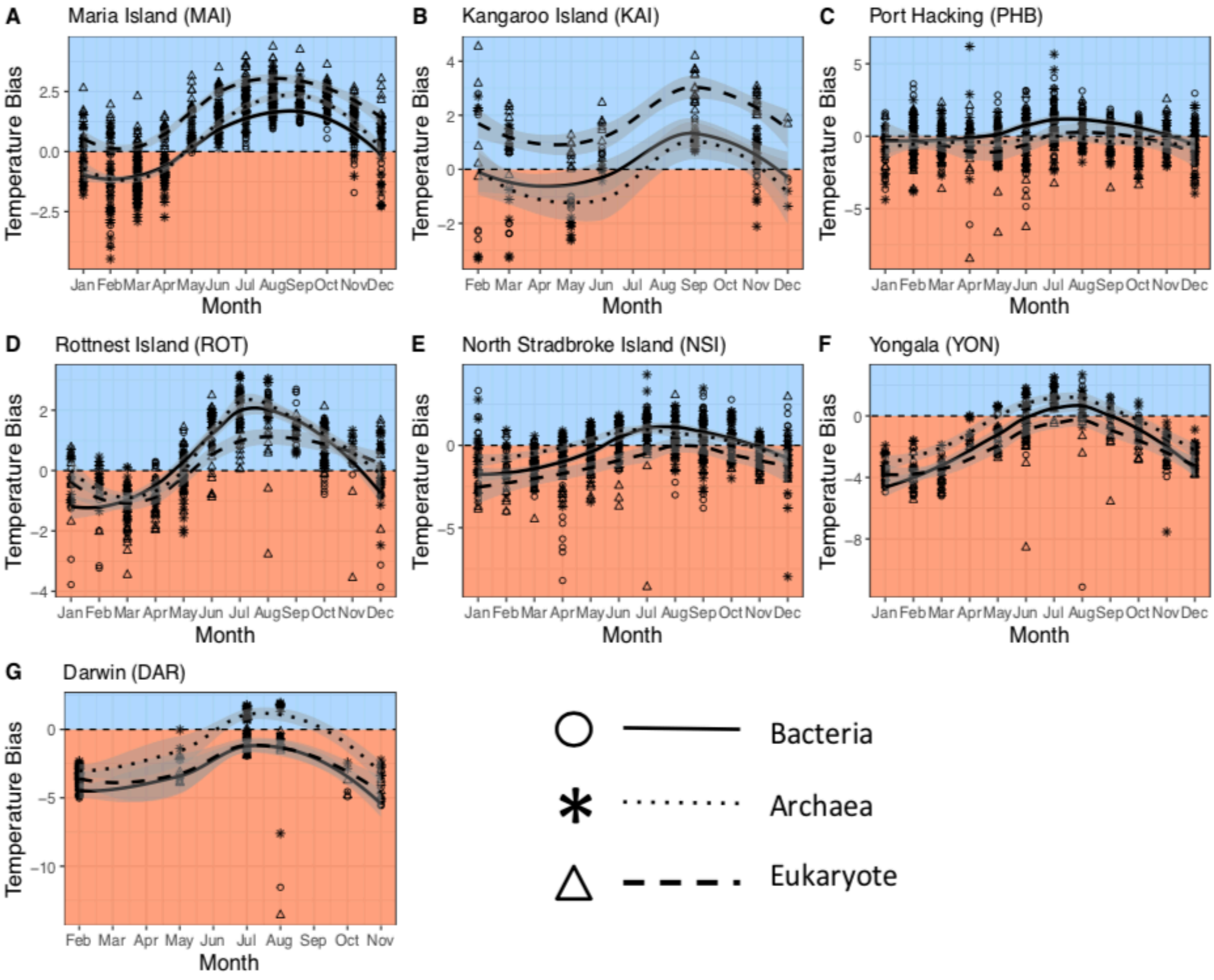

Supplementary Figure 5. Patterns of seasonal temperature bias (CTI – environmental temperature) at National Reference Stations. Communities with a positive thermal bias (blue shading) are composed of taxa with thermal optima warmer than environmental conditions, while communities with negative thermal bias are composed of taxa with thermal optima cooler than environmental conditions (red shading).

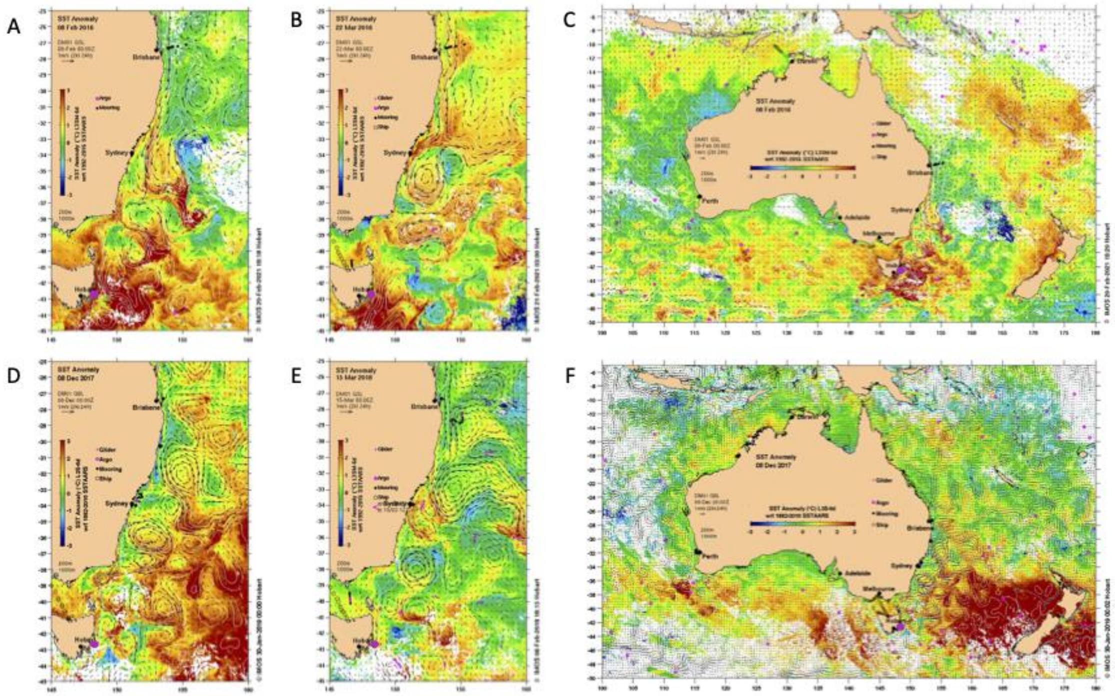

Supplementary Figure 6. Maps displaying SST anomalies during the 2015/16 MHW and 2017/18 MHW events in the Tasman Sea. Six-day composite night-only SST anomaly and current velocity images are centred around dates: A and C) Feb 8th, 2016; date of peak intensity of 2015/16 MHW at Maria Island National Reference Station (42° 35.80 S, 148° 14.00E), B) March 22nd, 2016; sampling date of highest observed microbial CTI during the 2015/16 MHW. D and F) Dec 8th, 2017; date of peak intensity of 2017/18 MHW at Maria Island National Reference Station (42° 35.80 S, 148° 14.00E), E) Mar 15th 2018; sampling date of highest observed microbial CTI during the 2017/18 MHW (Image source and interactive portal: oceancurrent.imos.org.au). Data was sourced from Australia's Integrated Marine Observing System (IMOS) – IMOS is enabled by the National Collaborative Research Infrastructure Strategy (NCRIS). It is operated by a consortium of institutions as an unincorporated joint venture, with the University of Tasmania as Lead Agent.

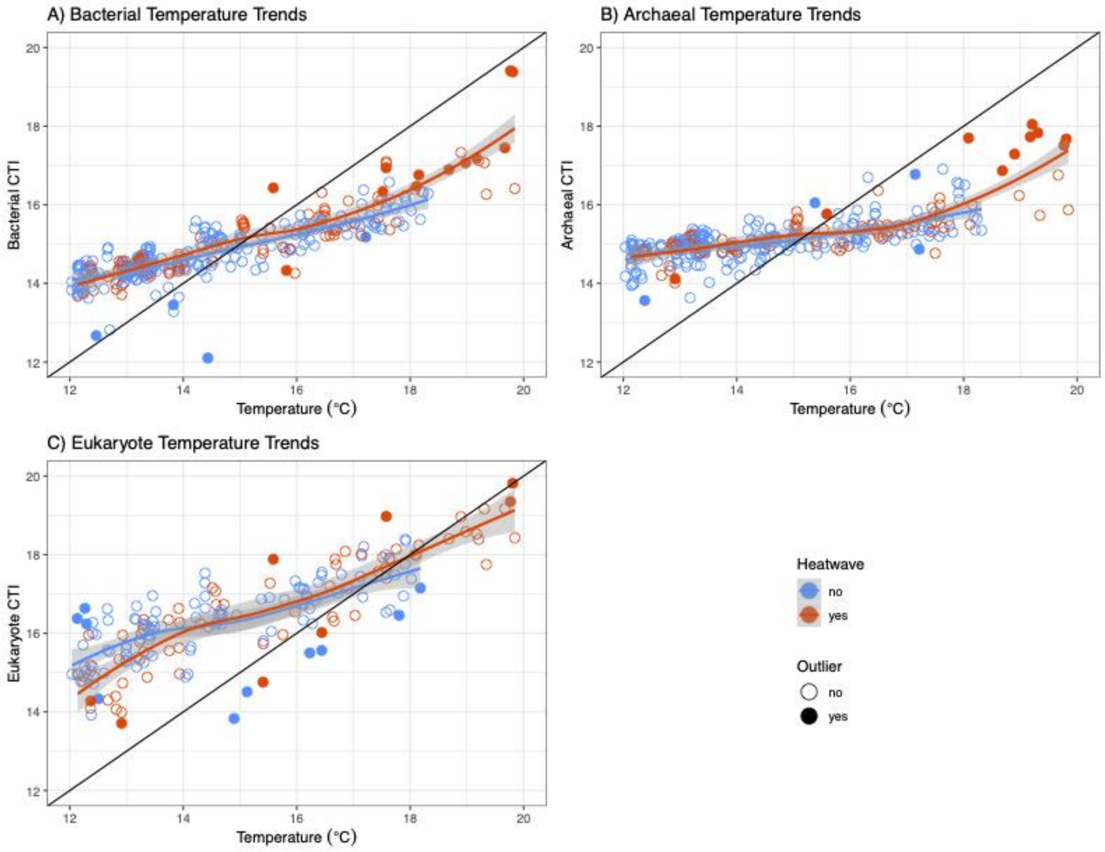

Supplementary Figure 7. Changes in CTI (CTI) in relation to *in situ* temperature at the Maria Island NRS in the Tasman Sea. Bacterial and archaeal assemblages sampled during MHW events (red) display a similar trend to those collected during non-MHW periods (blue) until temperatures exceed normal conditions (i.e., beyond temperatures experienced in non-MHW periods). Outliers were identified based on linear modelling of CTI : temperature at each depth individually. Loess smoothed trendlines across all depths displayed here are used to highlight where trends diverge.

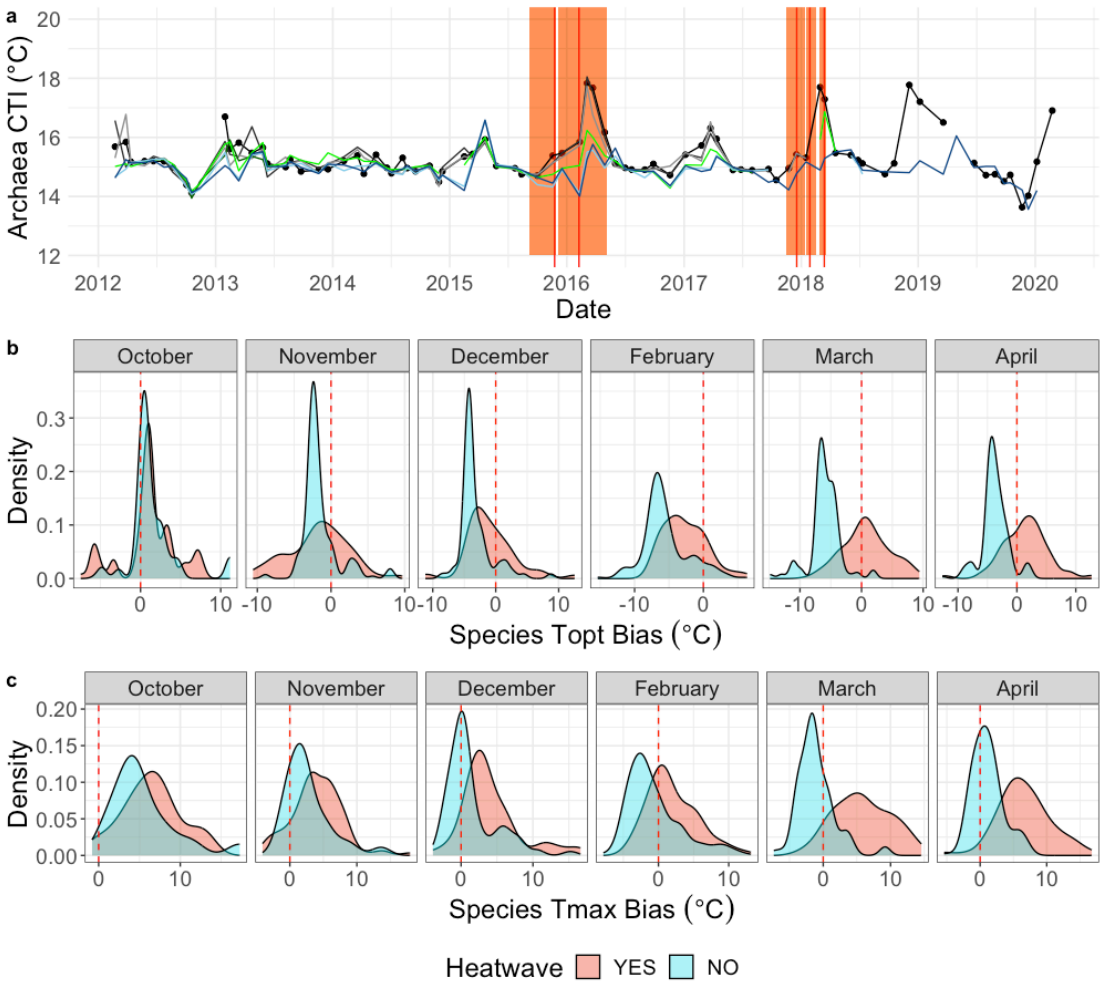

Supplementary Figure 8. The evolution of temperature selection for bacteria during the 2015/16 Tasman Sea MHW. A) Archaeal CTI (CTI) at the Maria Island NRS during the sampling period. Lines correspond to depths: Surface (black), 10m (dark gray), 20m (light gray), 40m (dark green) 50m (light green), 75m (light blue), 85m (dark blue). The 2015/16 and 2017/18 heatwave periods are shown as light red background, with peak intensity for each MHW event identified with a red bar. B) Density plots display the distribution of the thermal optima and C) the thermal maxima of archaea selected for (n= 620) or against (n = 499) in surface waters (0 and 10 m depth) during the 2015/16 MHW, compared to equivalent months during non-heatwave conditions (YES=selected for during the heatwave event, NO=selected against during the heatwave event). Dashed red line indicates zero bias. Notably, during February and March, the thermal maxima of many organisms selected against during the MHW is exceeded.

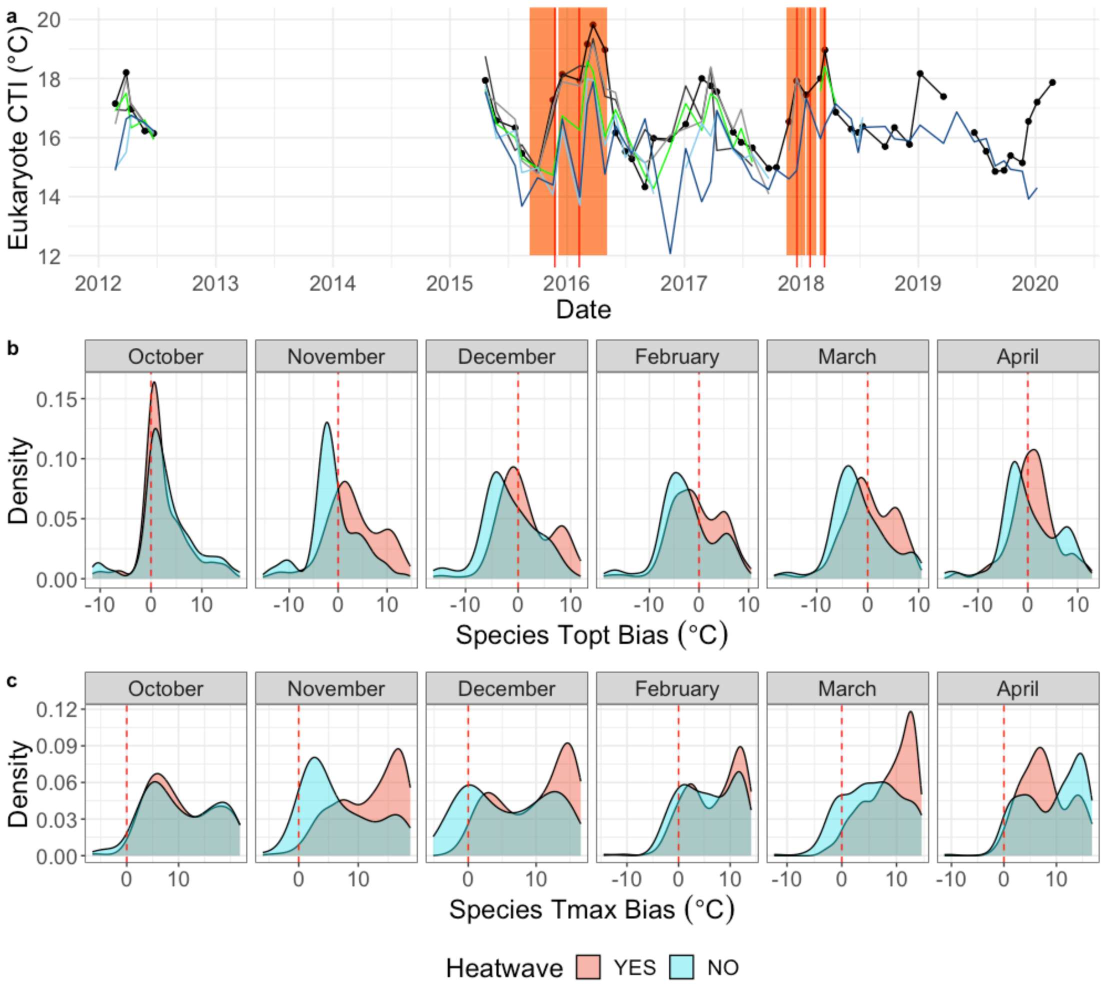

Supplementary Figure 9. The evolution of temperature selection for eukaryotes during the 2015/16 Tasman Sea MHW. A) Eukaryote CTI (CTI) at the Maria Island NRS during the sampling period. Lines correspond to depths: Surface (black), 10m (dark gray), 20m (light gray), 40m (dark green) 50m (light green), 75m (light blue), 85m (dark blue). The 2015/16 and 2017/18 heatwave periods are shown as light red background, with peak intensity for each MHW event identified with a red bar. B) Density plots display the distribution of the thermal optima and C) the thermal maxima of eukaryotes selected for (n = 2006) or against (n = 1423) in surface waters (0 and 10 m depth) during the 2015/16 MHW, compared to equivalent months during non-heatwave conditions (YES=selected for during the heatwave event, NO=selected against during the heatwave event). Dashed red line indicates zero bias. Notably, during February and March, the thermal maxima of many organisms selected against during the MHW is exceeded.

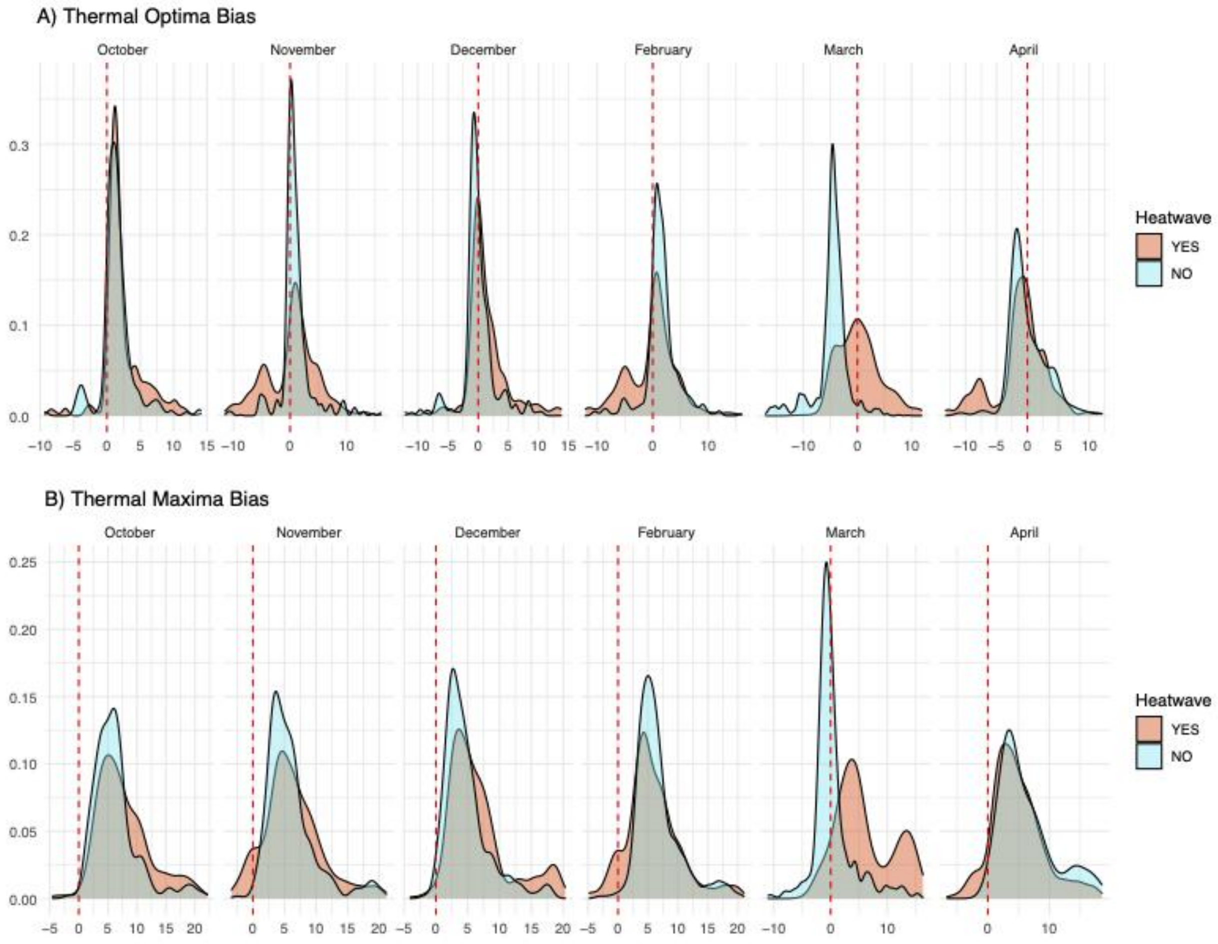

Supplementary Figure 10. Density plots display the distribution of a) the thermal optima and b) the thermal maxima of bacteria selected for (n = 2421) or against (n= 2124) in deep waters (75 and 85 m depth) during the 2015/16 MHW, compared to equivalent months during non-heatwave conditions.

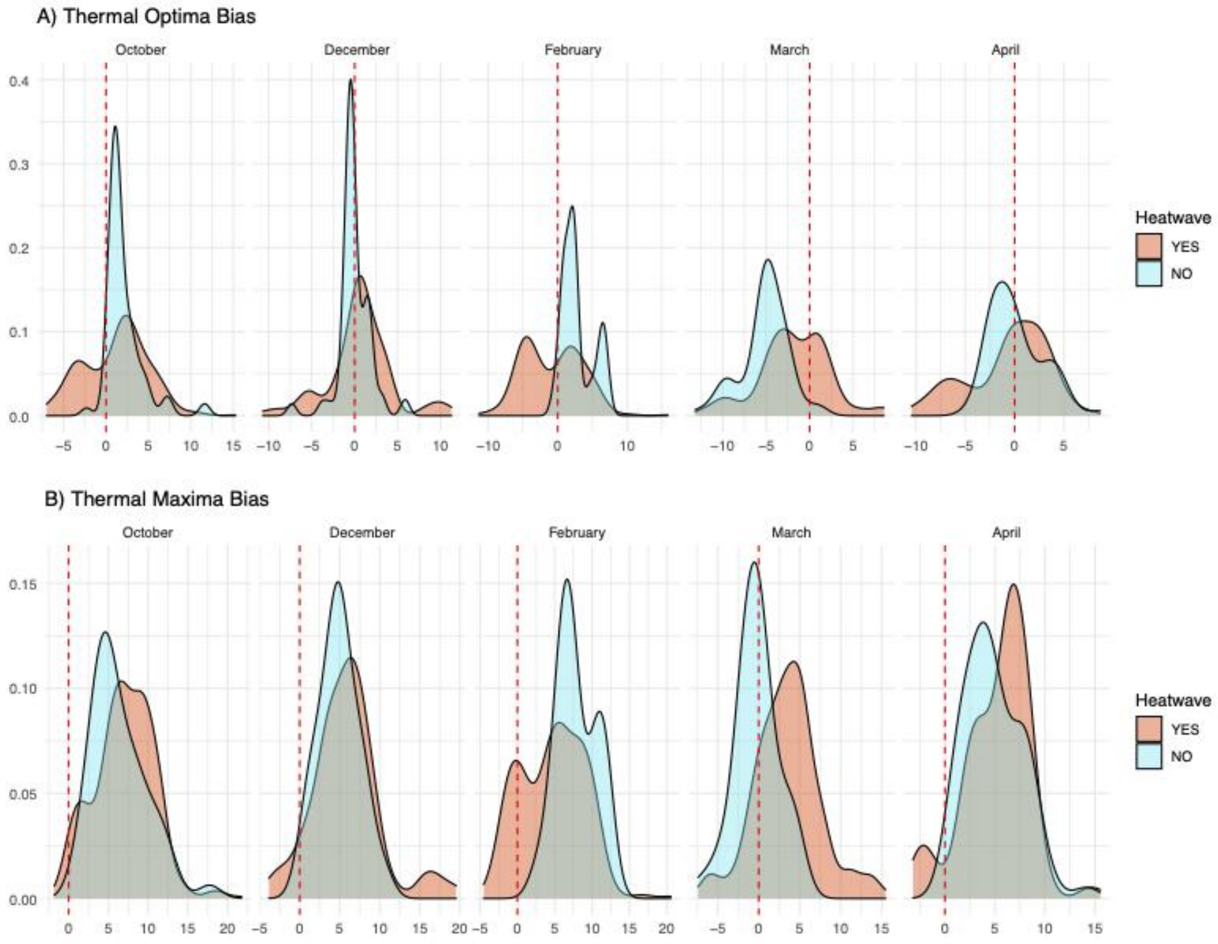

Supplementary Figure 11. Density plots display the distribution of a) the thermal optima and b) the thermal maxima of archaea selected for (n = 813) or against (n = 227) in deep waters (75 and 85 m depth) during the 2015/16 MHW, compared to equivalent months during non-heatwave conditions.

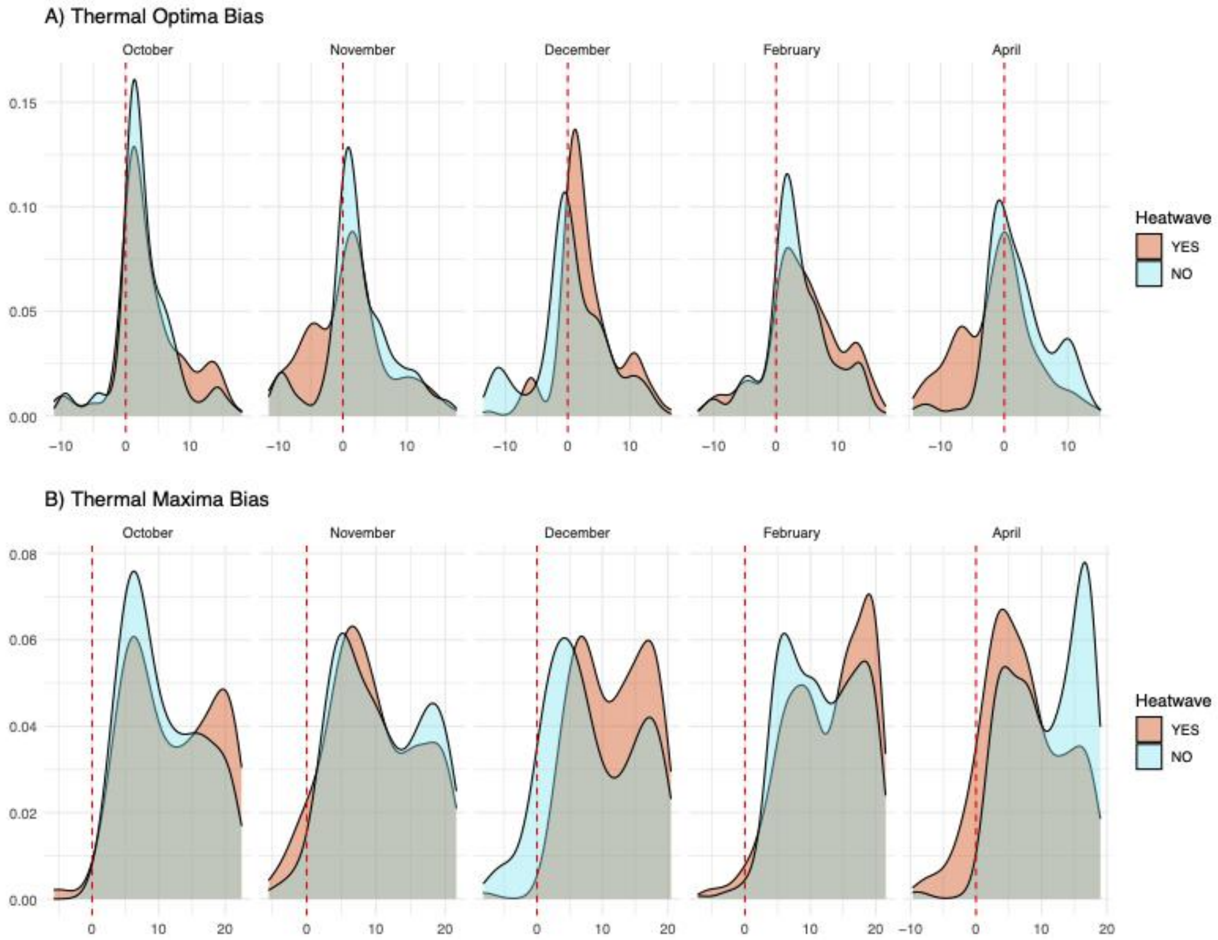

Supplementary Figure 12. Density plots display the distribution of a) the thermal optima and b) the thermal maxima of eukaryotes selected for (n = 2189) or against (n = 1815) in deep waters (75 and 85 m depth) during the 2015/16 MHW, compared to equivalent months during non-heatwave conditions.

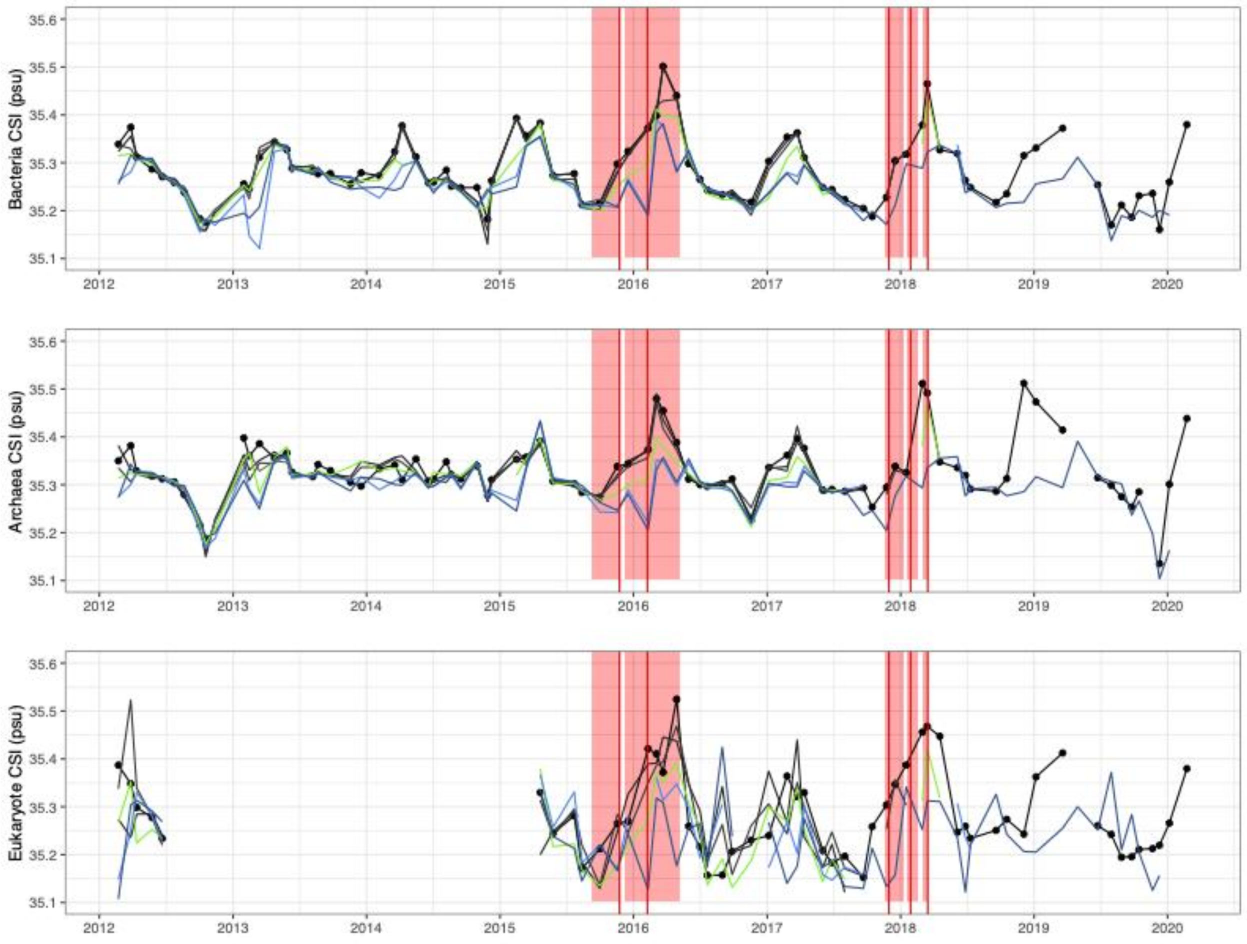

Supplementary Figure 13. CSI (CSI) of bacteria, archaea and eukaryote communities at the Maria Island NRS during the sampling period. Lines correspond to depths: Surface (black), 10m (gray), 50m (green), 75m (lightblue), 85m (dark blue).The 2015/16 and 2017/18 heatwave periods are shown as light red background, with peak intensity for each MHW identified with a red bar.

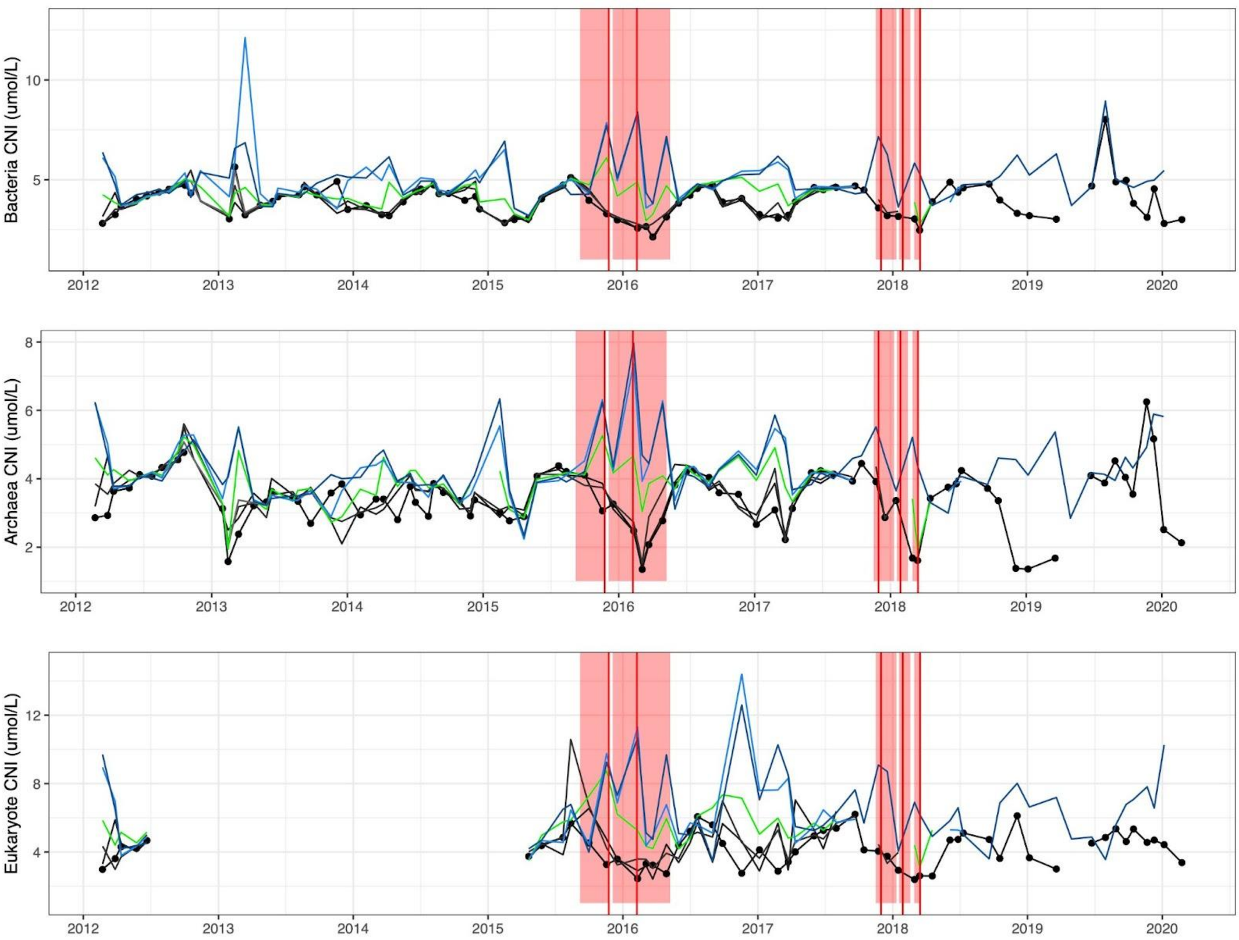

Supplementary Figure 14. Community nitrogen index (CNI) of bacteria, archaea and eukaryote communities at the Maria Island NRS during the sampling period. Lines correspond to depths: Surface (black), 10m (gray), 50m (green), 75m (lightblue), 85m (dark blue).The 2015/16 and 2017/18 heatwave periods are shown as light red background, with peak intensity for each MHW identified with a red bar.

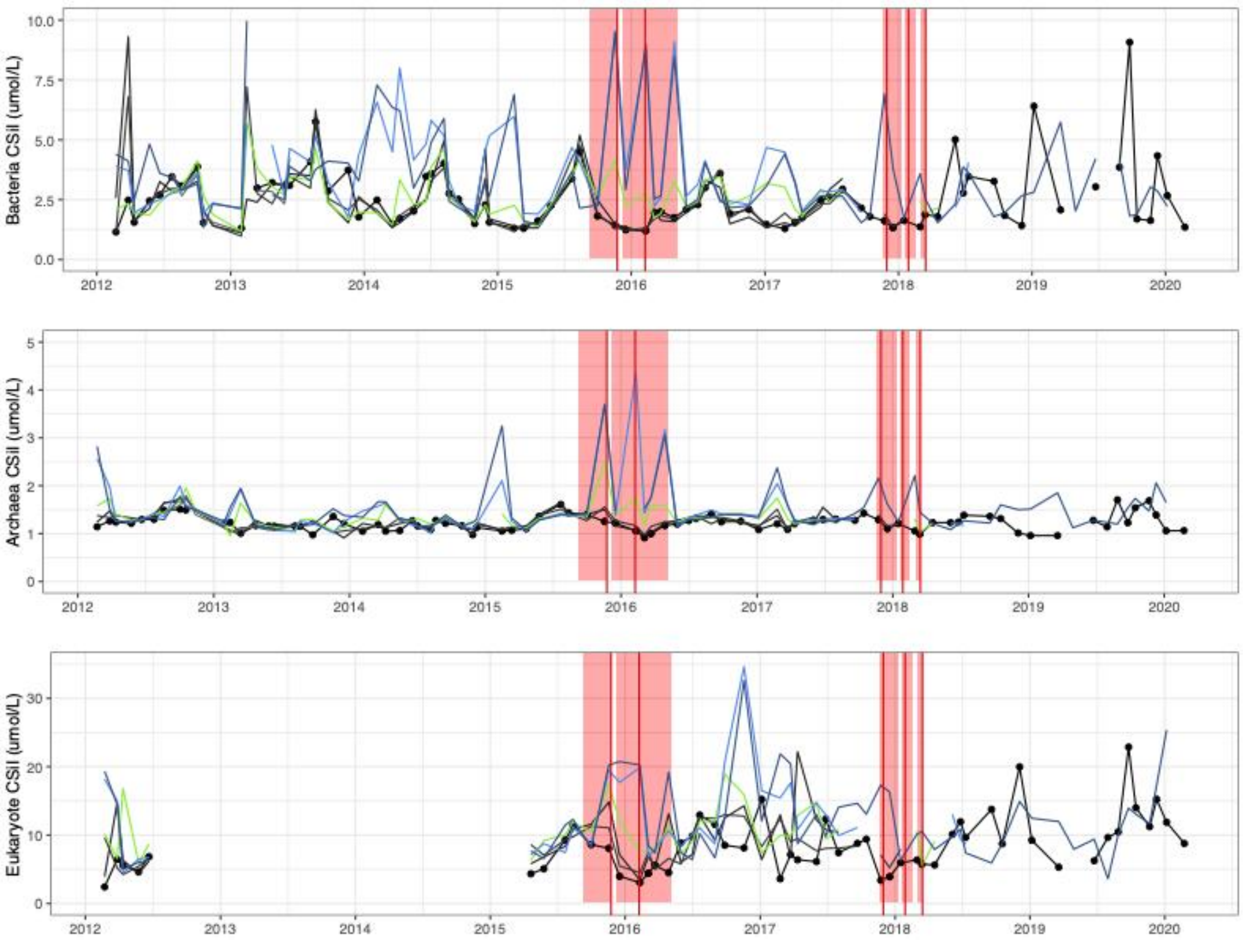

Supplementary Figure 15. CSiI (CSiI) of bacteria, archaea and eukaryote communities at the Maria Island NRS during the sampling period. Lines correspond to depths: Surface (black), 10m (gray), 50m (green), 75m (lightblue), 85m (dark blue).The 2015/16 and 2017/18 heatwave periods are shown as light red background, with peak intensity for each MHW identified with a red bar.

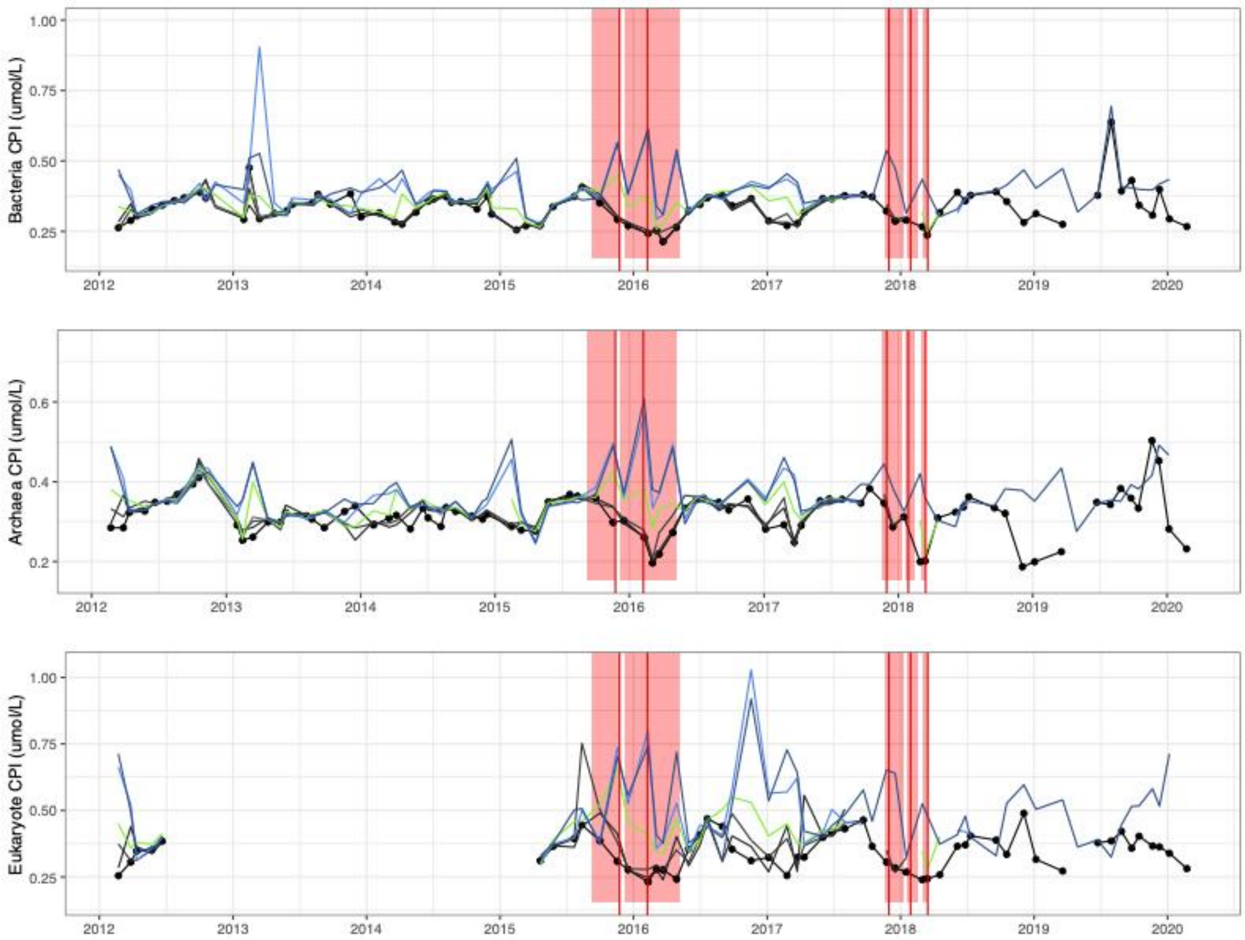

Supplementary Figure 16. CPI (CPI) of bacteria, archaea and eukaryote communities at the Maria Island NRS during the sampling period. Lines correspond to depths: Surface (black), 10m (gray), 50m (green), 75m (lightblue), 85m (dark blue).The 2015/16 and 2017/18 heatwave periods are shown as light red background, with peak intensity for each MHW identified with a red bar.

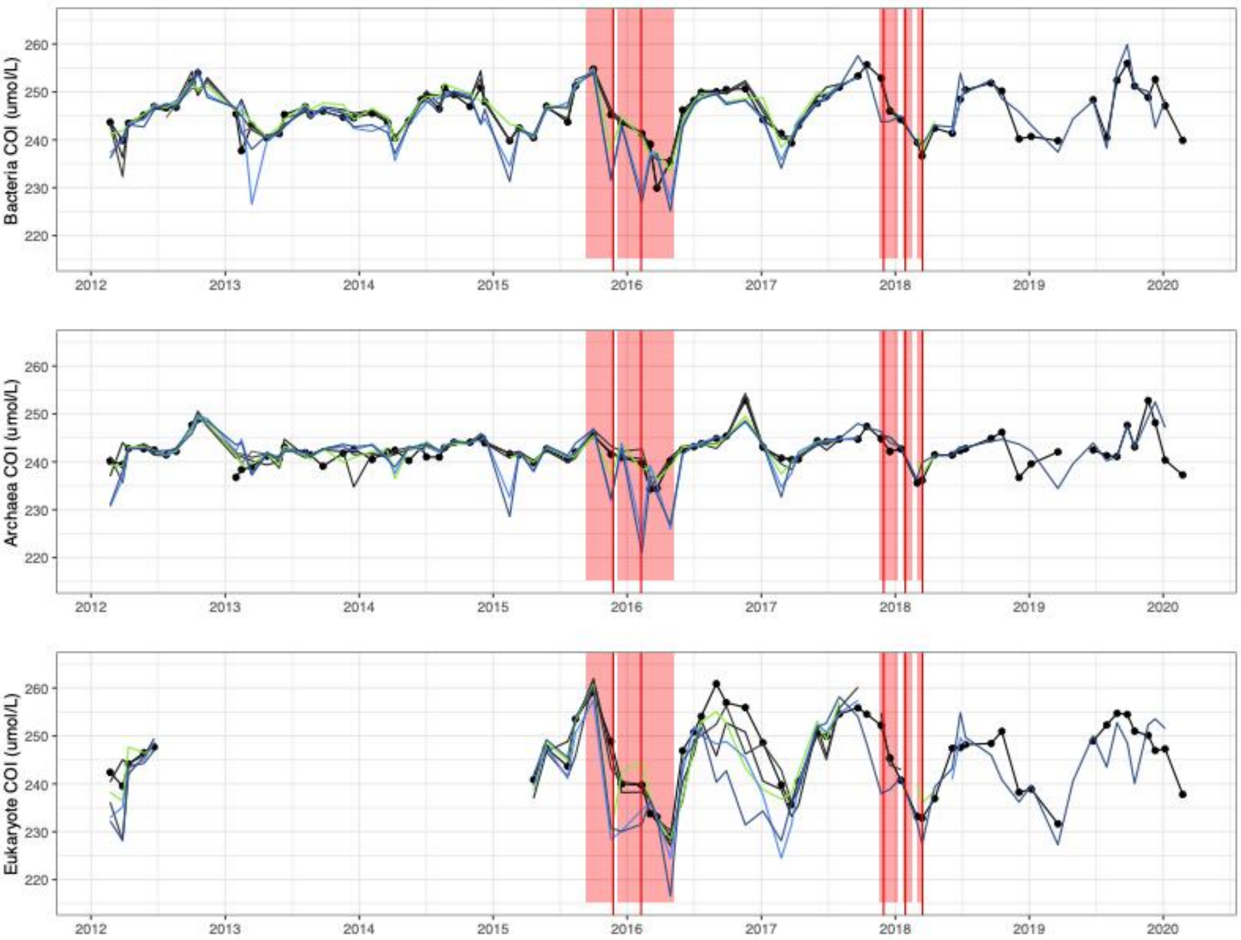

Supplementary Figure 17. COI (COI) of bacteria, archaea and eukaryote communities at the Maria Island NRS during the sampling period. Lines correspond to depths: Surface (black), 10m (gray), 50m (green), 75m (lightblue), 85m (dark blue).The 2015/16 and 2017/18 heatwave periods are shown as light red background, with peak intensity for each MHW identified with a red bar.

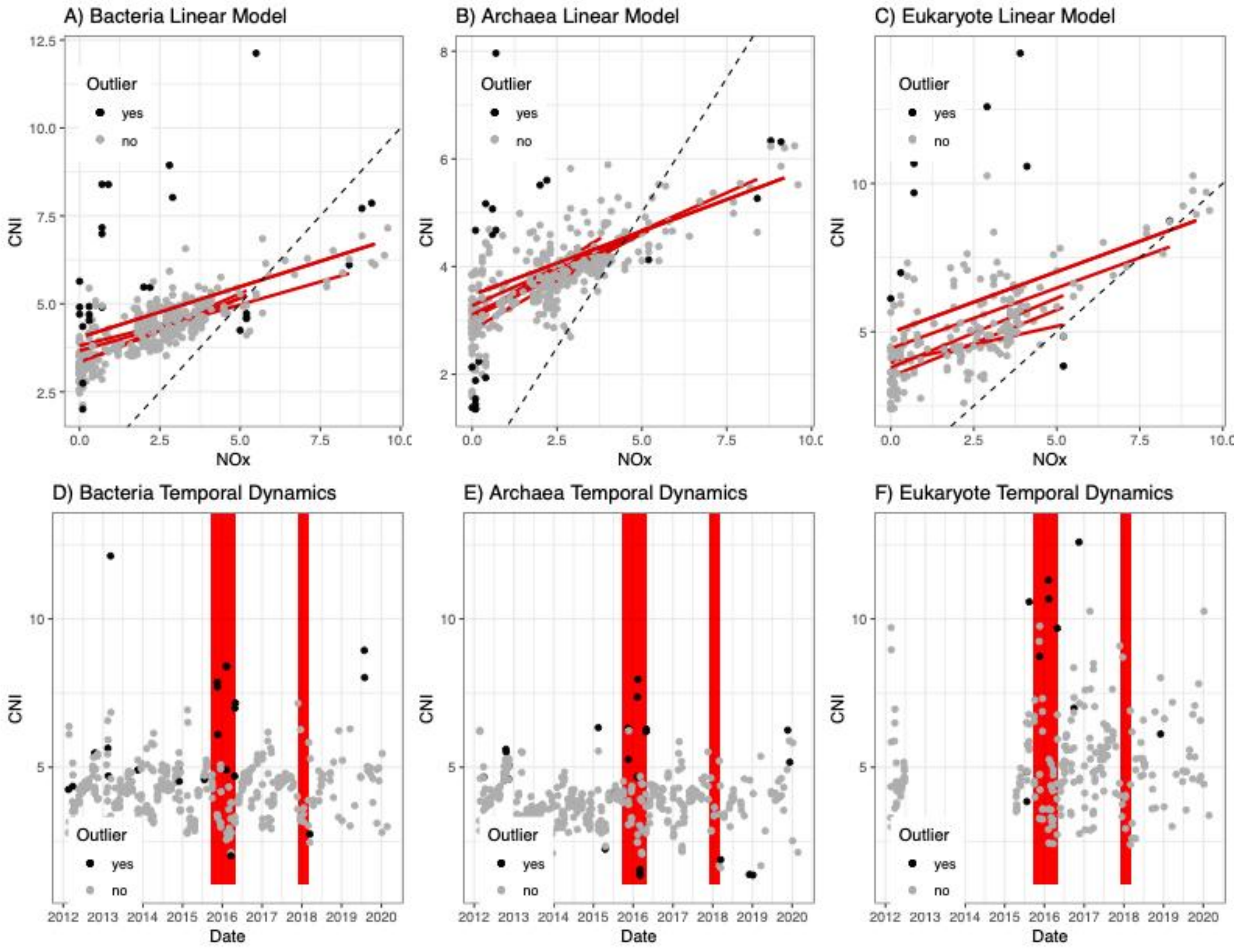

Supplementary Figure 18. A-C) Relationships between community weighted nitrate + nitrite index (CNI) and *in situ* NOx measured at Maria Island NRS at the time of sampling. Dashed black line represents 1:1. Smoothed lines represent linear models calculated at each depth. D-F) Temporal dynamics of CNI at Maria Island NRS. Periods of MHW activity during 2015/16 and 2017/18 are outlined with red boxes.

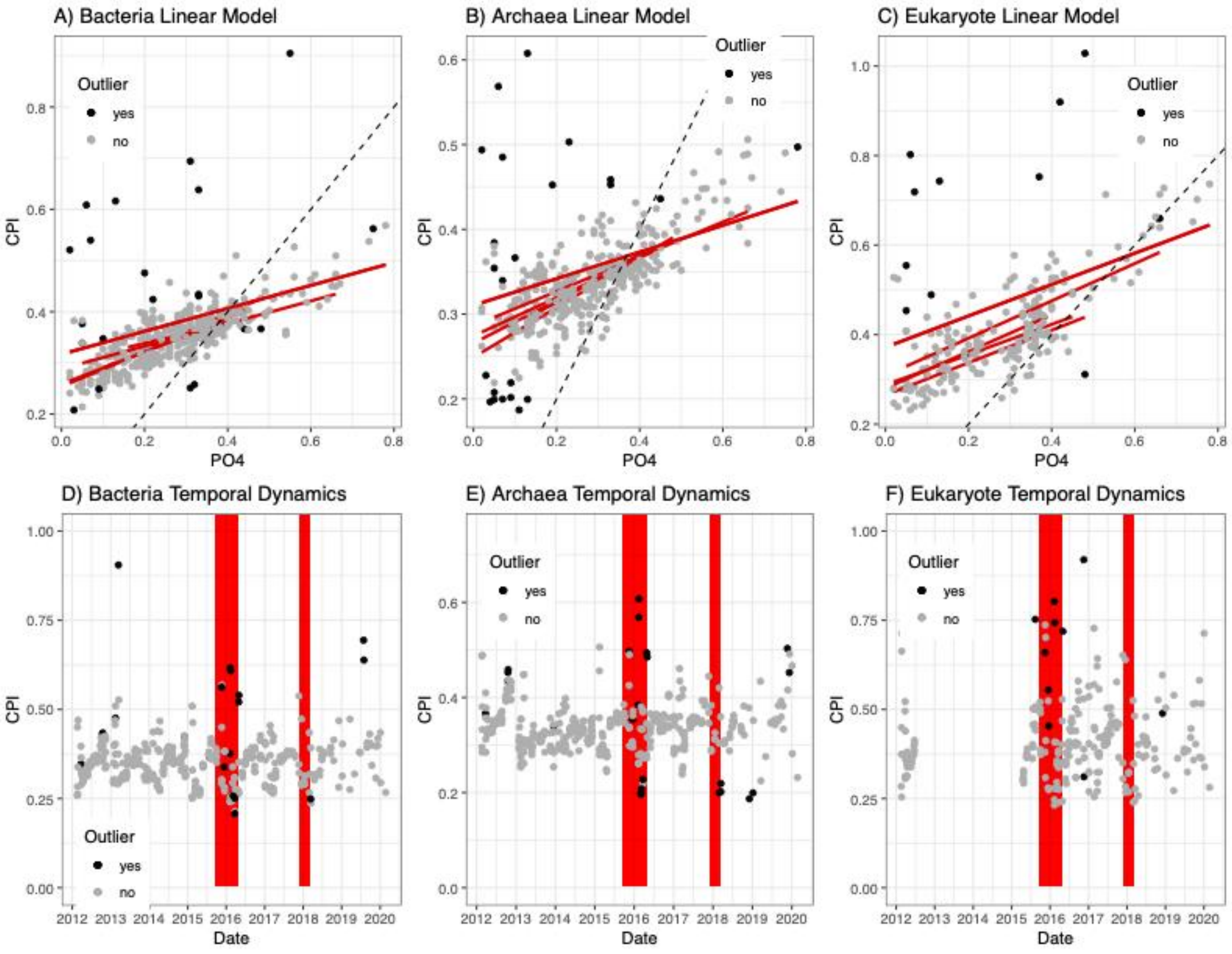

Supplementary Figure 19. A-C) Relationships between community weighted PO4 index (CPI) and *in situ* PO4 measured at Maria Island NRS at the time of sampling. Dashed black line represents 1:1. Smoothed lines represent linear models calculated at each depth. D-F) Temporal dynamics of CNI at Maria Island NRS. Periods of MHW activity during 2015/16 and 2017/18 are outlined with red boxes.

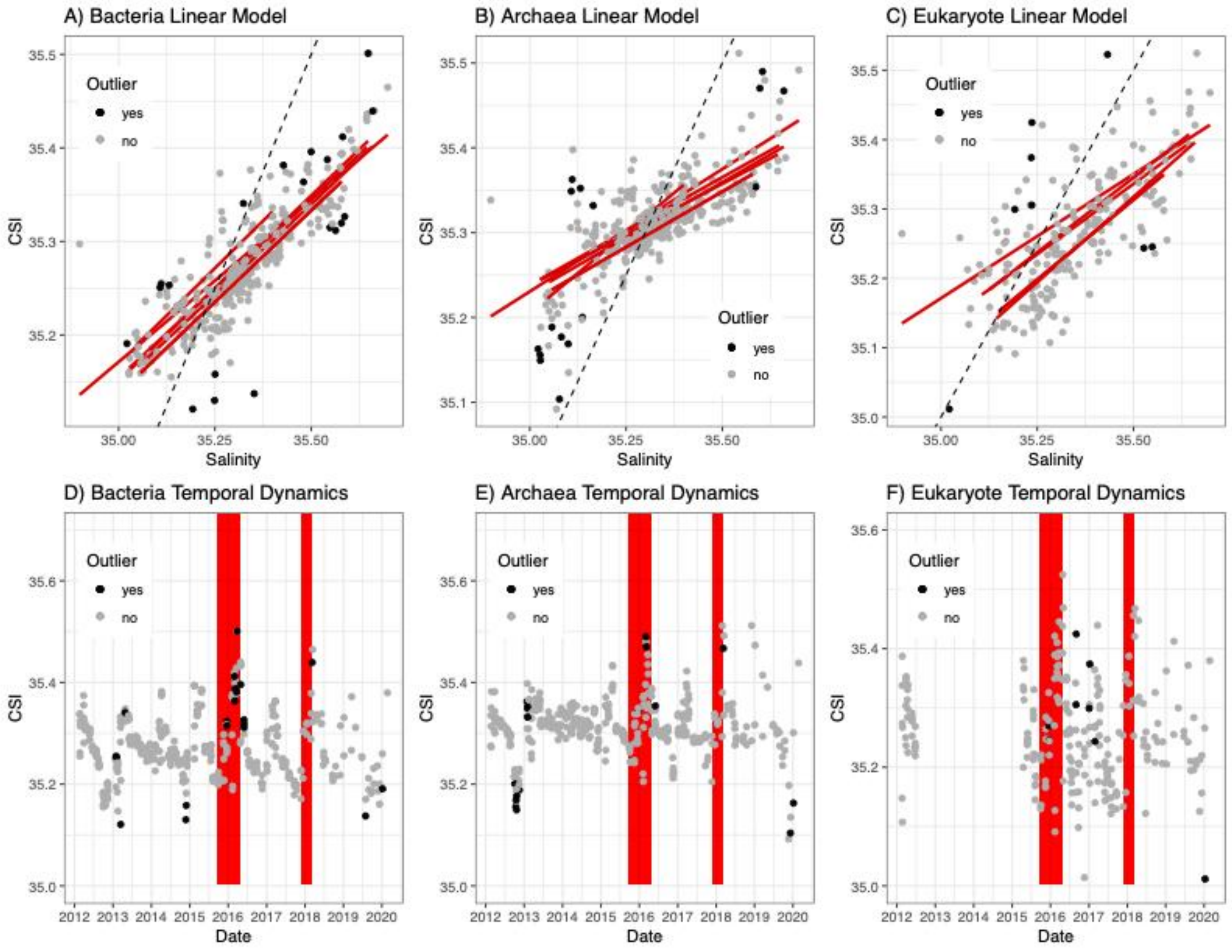

Supplementary Figure 20. A-C) Relationships between community weighted salinity index (CSI) and *in situ* salinity measured at Maria Island NRS at the time of sampling. Dashed black line represents 1:1. Smoothed lines represent linear models calculated at each depth. D-F) Temporal dynamics of CNI at Maria Island NRS. Periods of MHW activity during 2015/16 and 2017/18 are outlined with red boxes.

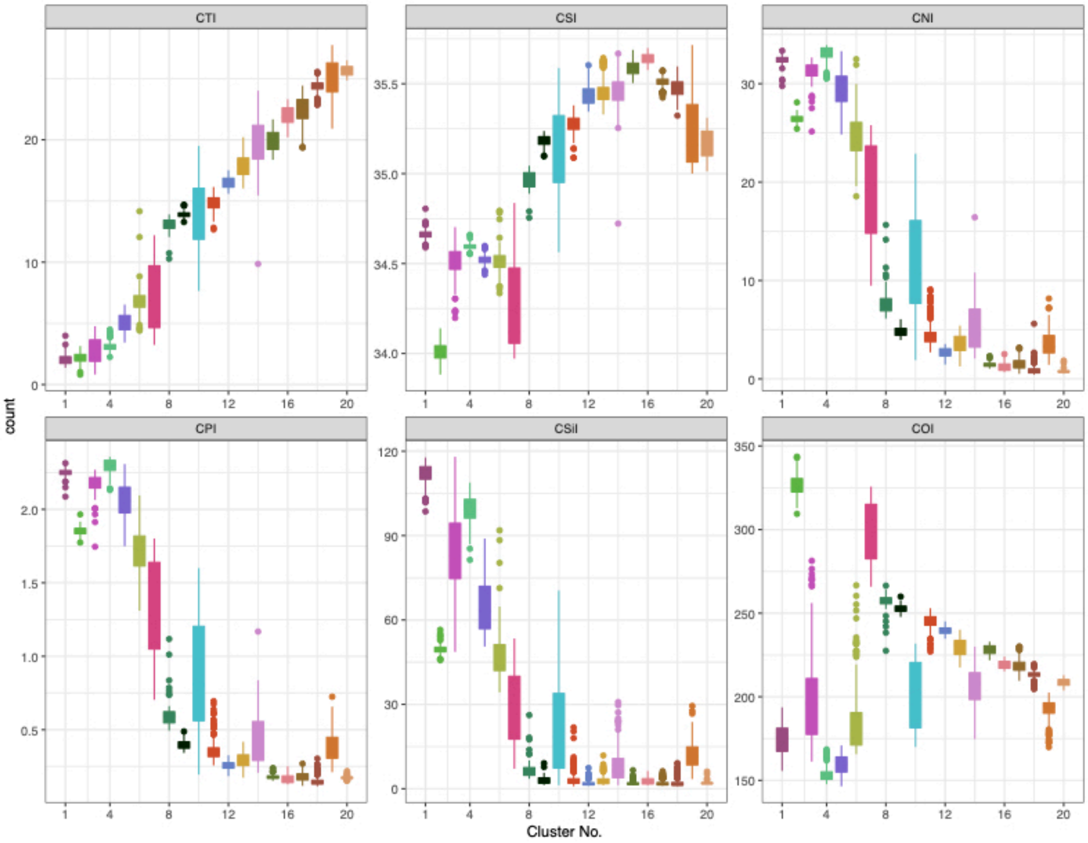

Supplementary Figure 21. Relationship between bacterial clusters and indices reflective of the environmental niche characteristics of the assemblage.

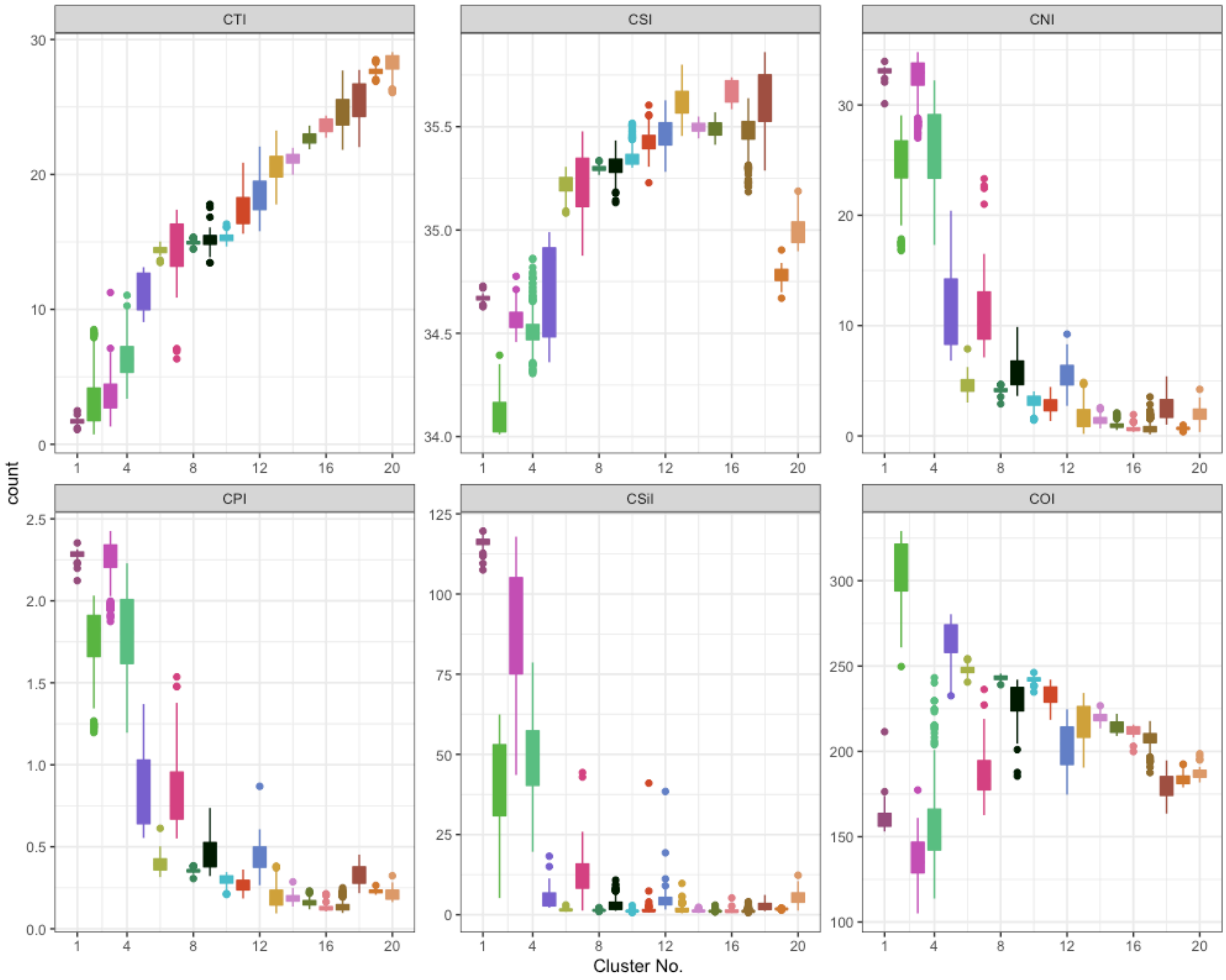

Supplementary Figure 22. Relationship between archaeal clusters and indices reflective of the environmental niche characteristics of the assemblage.

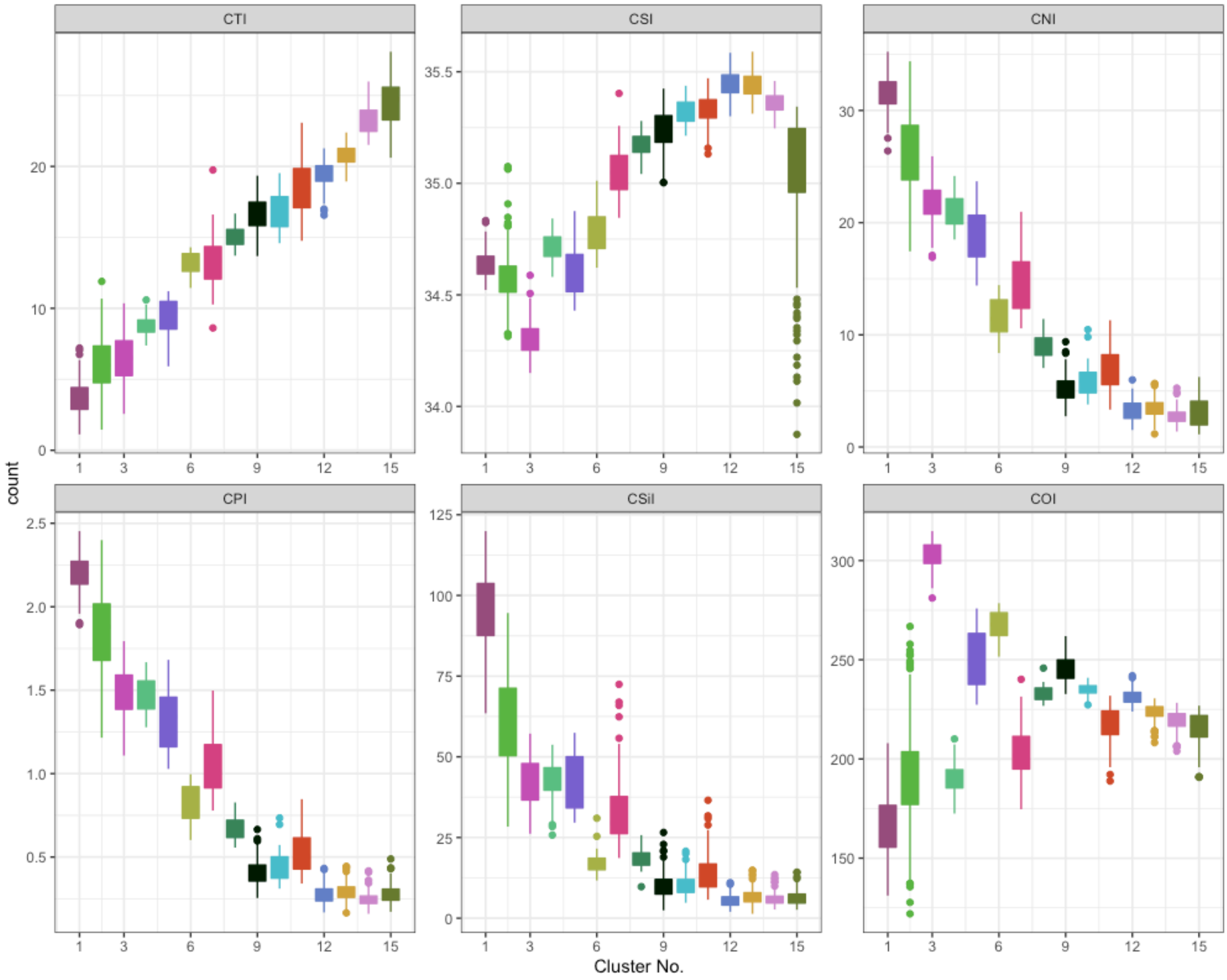

Supplementary Figure 23 Relationship between eukaryote clusters and indices reflective of the environmental niche characteristics of the assemblage.

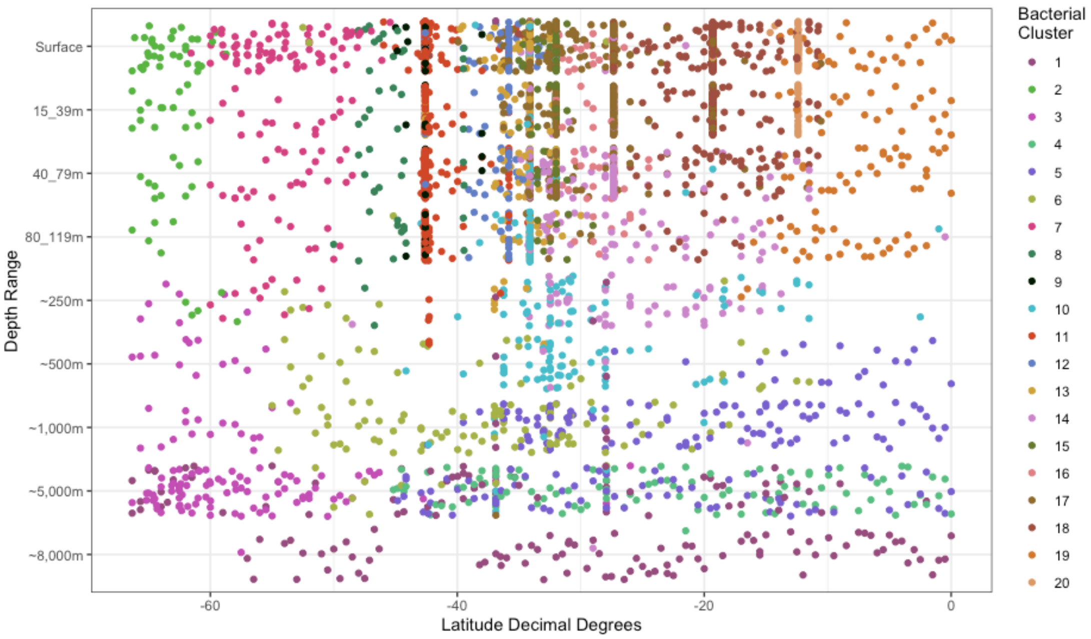

Supplementary Figure 24. Depth and latitude of each sample in the dataset coloured according to the bacterial cluster they belong to based on community niche characteristics.

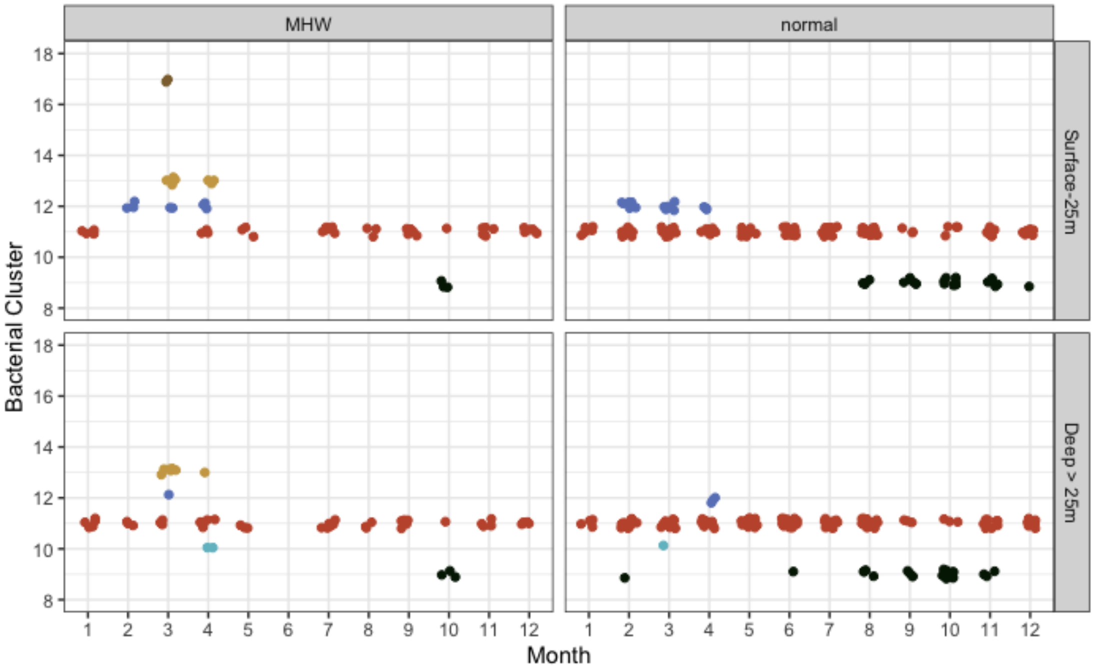

Supplementary Figure 25. Cluster state of bacterial assemblages during MHW conditions and equivalent months during non-heatwave conditions at Maria Island. During “normal conditions there is a repeatable seasonal pattern in clusters appearance. During MHW conditions several new clusters appear.

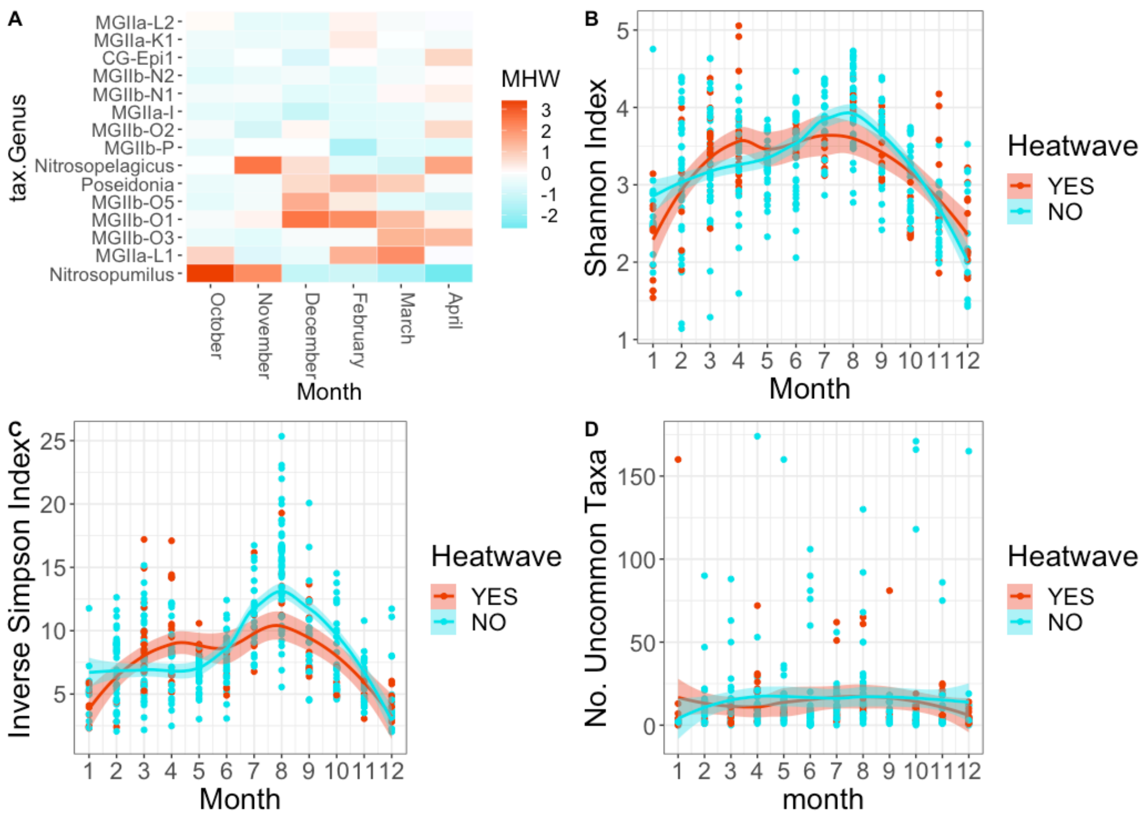

Supplementary Figure 26. Marine heatwaves lead to compositional and structural changes in microbial assemblages. a) Heatmap detailing the archaeal genera contributing most to the compositional difference between surface samples collected in equivalent months during MHW and non-MHW conditions. b) The seasonal cycle of archaeal Shannon diversity, c) Inverse Simpsons diversity and d) the number of "uncommon" archaeal taxa in each sample are all modulated by MHW condition. Samples collected during MHW conditions (n=106) are in red and those collected during non-MHW conditions (n=384) are in blue.

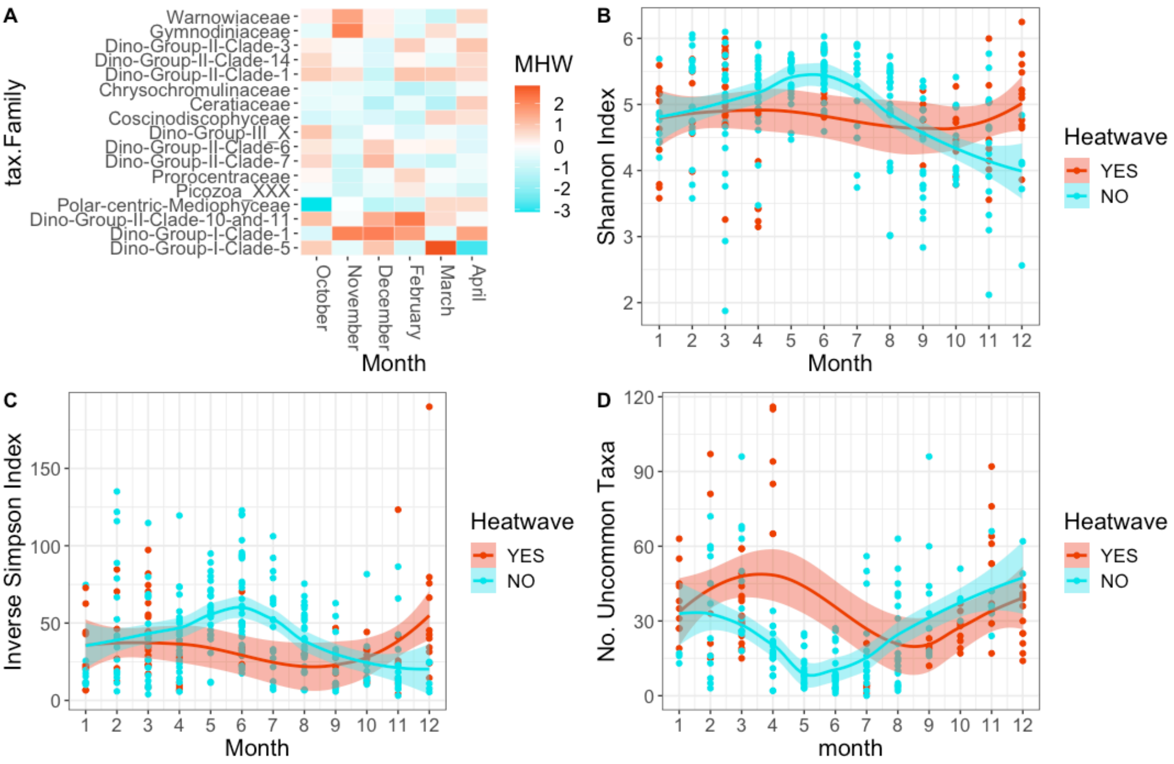

Supplementary Figure 27. Marine heatwaves lead to compositional and structural changes in microbial assemblages. a) Heatmap detailing the eukaryotic genera contributing most to the compositional difference between surface samples collected in equivalent months during MHW and non-MHW conditions. b) The seasonal cycle of eukaryotic Shannon diversity, c) Inverse Simpsons diversity and d) the number of "uncommon" eukaryotic taxa in each sample are all modulated by MHW condition. Samples collected during MHW conditions (n=80) are in red and those collected during non-MHW conditions (n=222) are in blue.

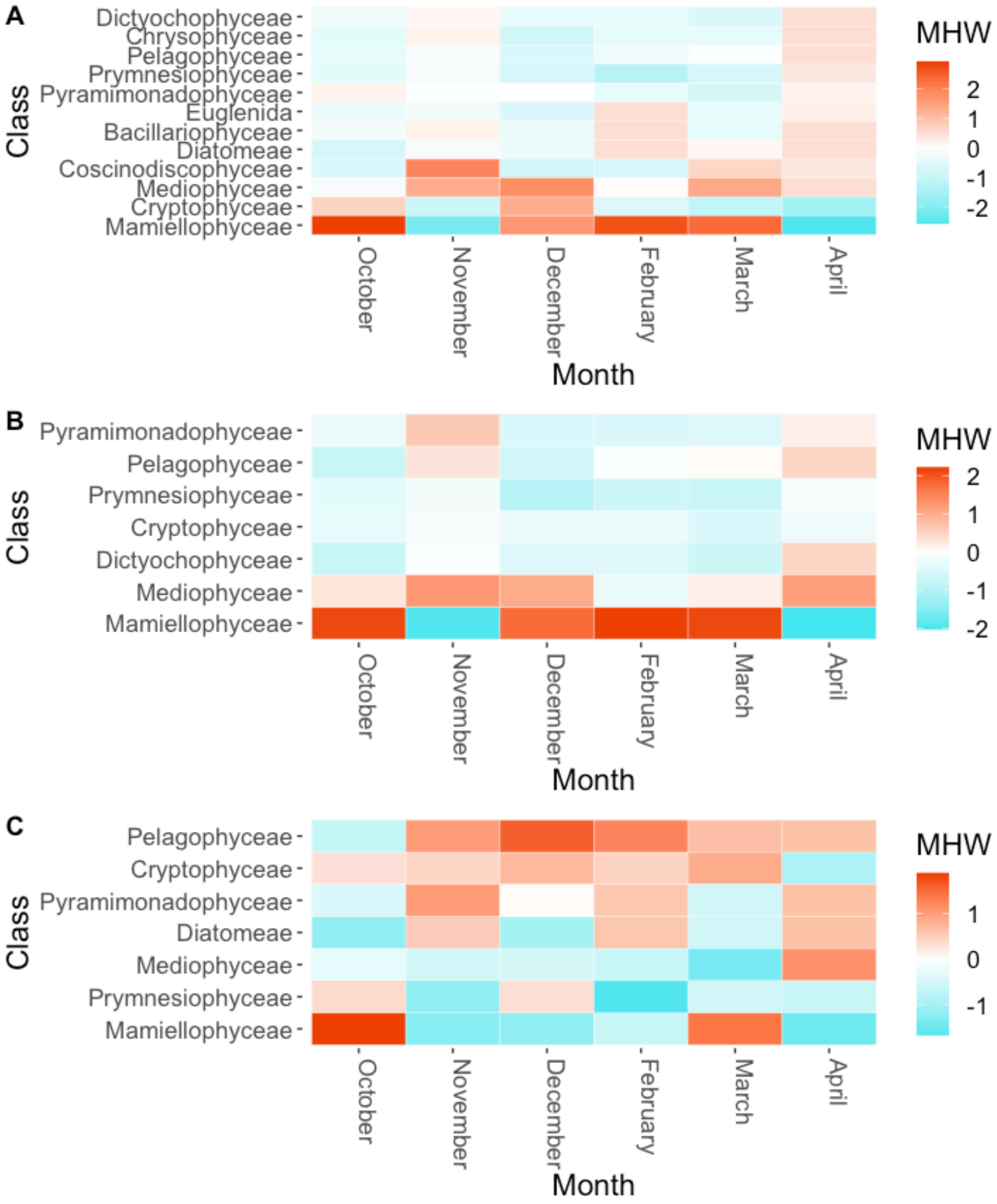

Supplementary Figure 28. Heatmaps of eukaryotic phytoplankton, identified using chloroplast 16S rRNA gene sequences from the bacterial dataset, selected for or against during the 2015/16 marine heatwave. A) Surface (0-10m), B0 Mid (25-50m) and C) deep (75-85m)

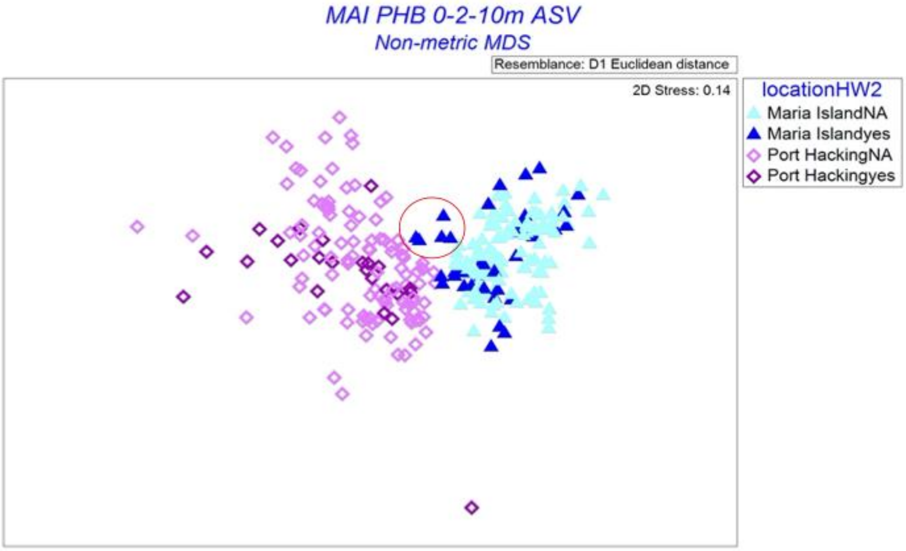

Supplementary Figure 29. Non-metric multidimensional scaling plot based on Bray-Curtis transformed similarity matrix of surface assemblages (0,10m) sampled at Maria Island and Port Hacking National Reference Stations. Colours denote station of origin and MHW status (NA = no marine heatwave, YES = marine heatwave). Red circle denotes relevant surface samples from the 2015/16 MHW at Maria Island.

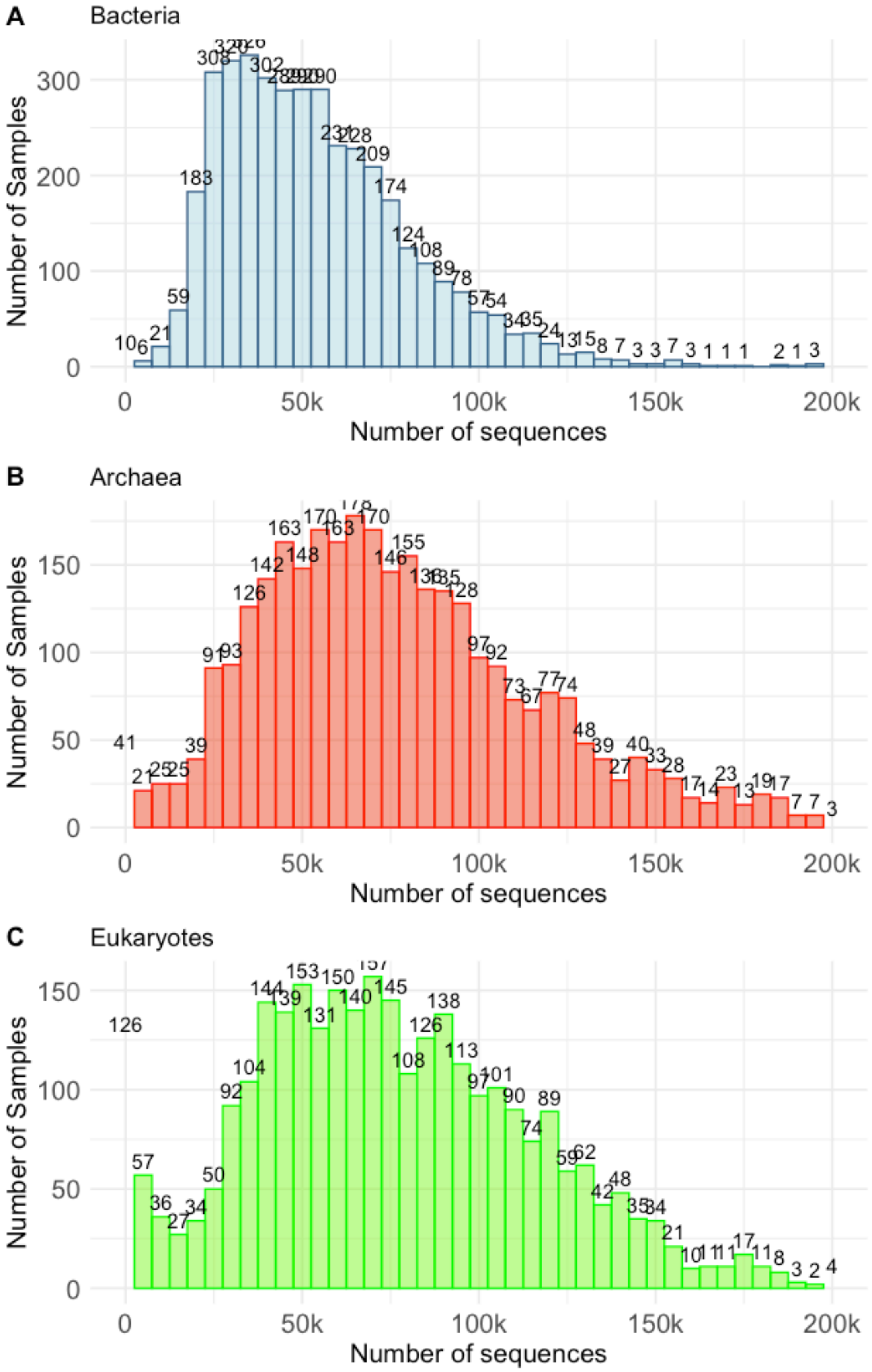

Supplementary Figure 30. Distribution of number of sequences per sample in the dataset for A) Bacteria, B) Archaea and C) Eukaryotes. Histograms were cut off at 200k for presentation, removing 10, 62 and 43 samples with > 200,000 sequences from A, B, and C respectively.

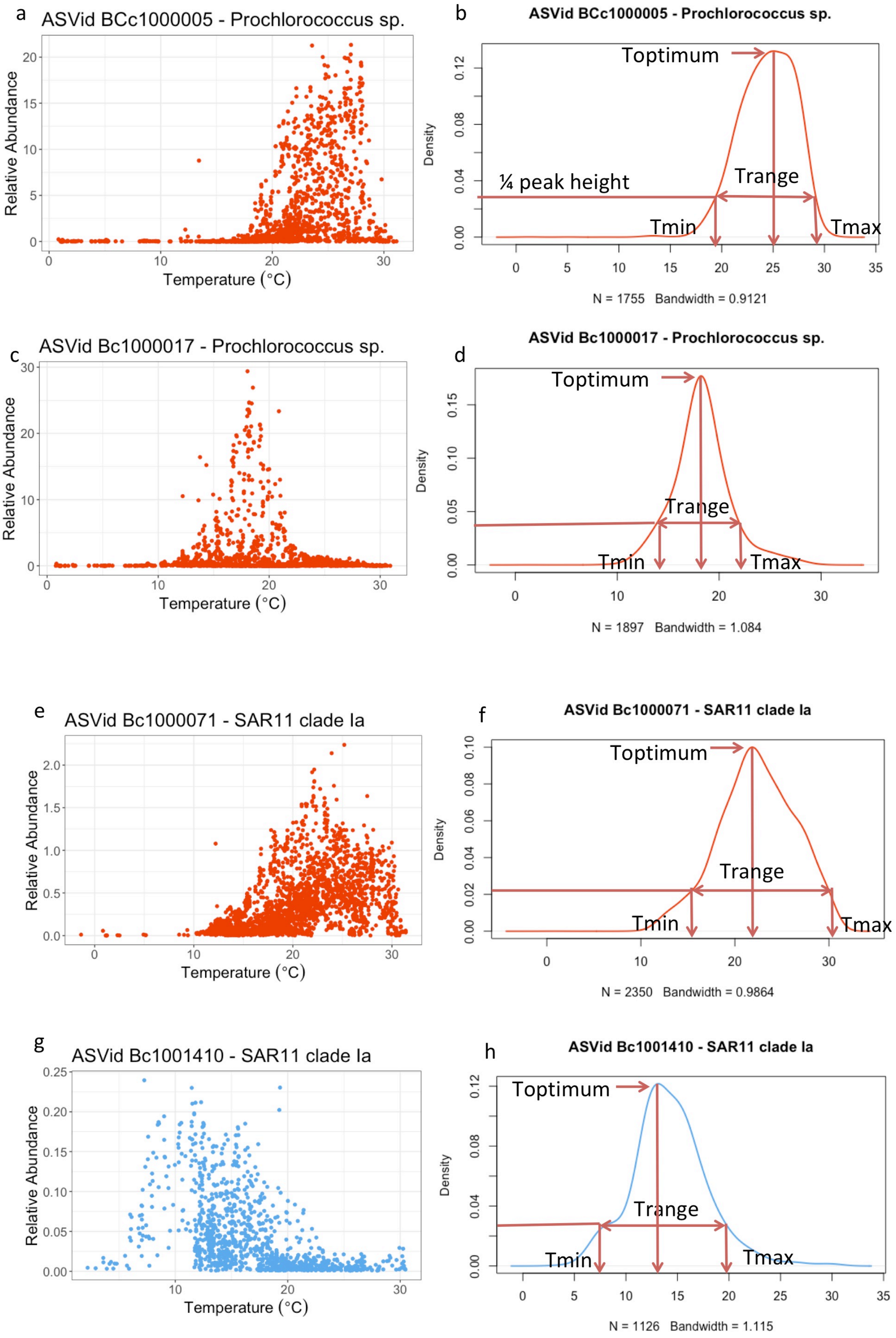

a
ASVid BCc1000005 - Prochlorococcus sp.
Relative Abundance
Temperature (°C)
b
ASVid BCc1000005 - Prochlorococcus sp.
Density
Toptimum
¼ peak height
Trange
Tmin
Tmax
N = 1755 Bandwidth = 0.9121
c
ASVid Bc1000017 - Prochlorococcus sp.
Relative Abundance
Temperature (°C)
d
ASVid Bc1000017 - Prochlorococcus sp.
Density
Toptimum
Trange
Tmin
Tmax
N = 1897 Bandwidth = 1.084
e
ASVid Bc1000071 - SAR11 clade Ia
Relative Abundance
Temperature (°C)
f
ASVid Bc1000071 - SAR11 clade Ia
Density
Toptimum
Trange
Tmin
Tmax
N = 2350 Bandwidth = 0.9864
g
ASVid Bc1001410 - SAR11 clade Ia
Relative Abundance
Temperature (°C)
h
ASVid Bc1001410 - SAR11 clade Ia
Density
Toptimum
Trange
Tmin
Tmax
N = 1126 Bandwidth = 1.115

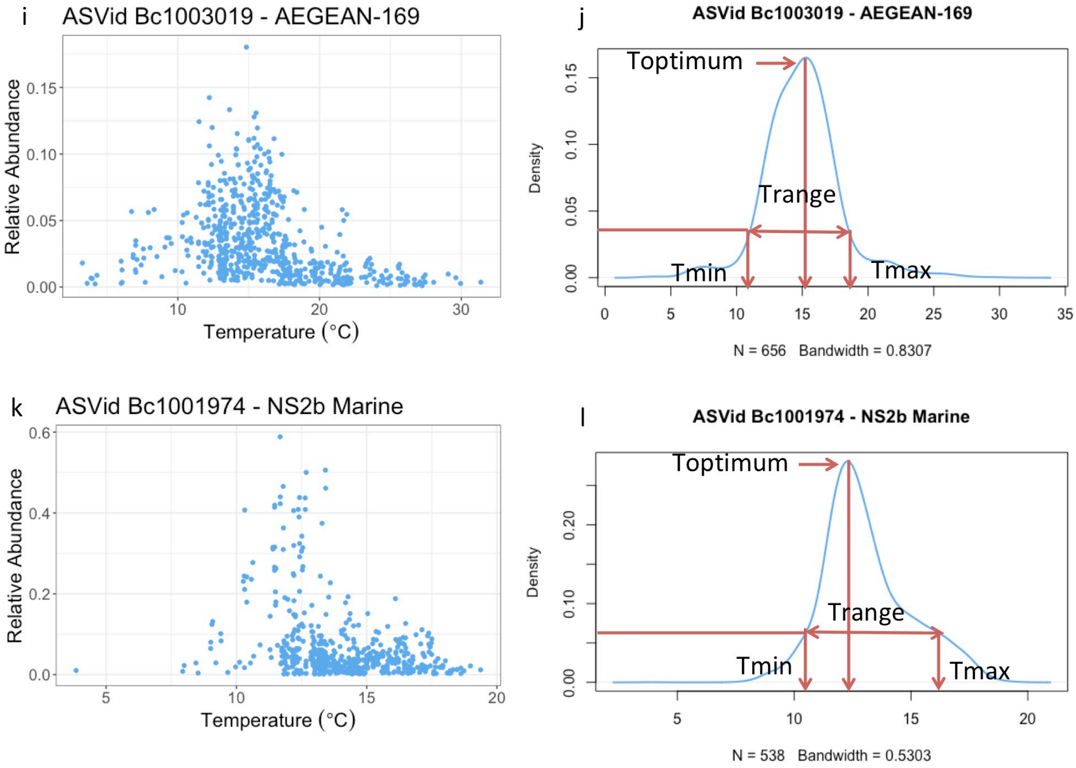

Supplementary Figure 31. Example data and respective kernel density (KD) models of ASVs that were selected for (red) and against (blue) during MHW conditions. a,c,e,g,i,k) Relative abundance of select ASVs along temperature gradients in the entire dataset. b,d,f,h,j,l) Respective kernel density models indicating Toptimum (referred to as the species temperature index), Tmin = temperature minimum, (1/4 KD peak height left); Tmax = temperature maximum (1/4 peak height right); Trange = temperature range (Tmin- Tmax). N = number of observations used in the KD model. Bandwith was selected using "rule of thumb" approach (parameter bw="nrd0".) Models were initiated 3 °C either side of the minimum and maximum temperatures at which each ASV was observed.

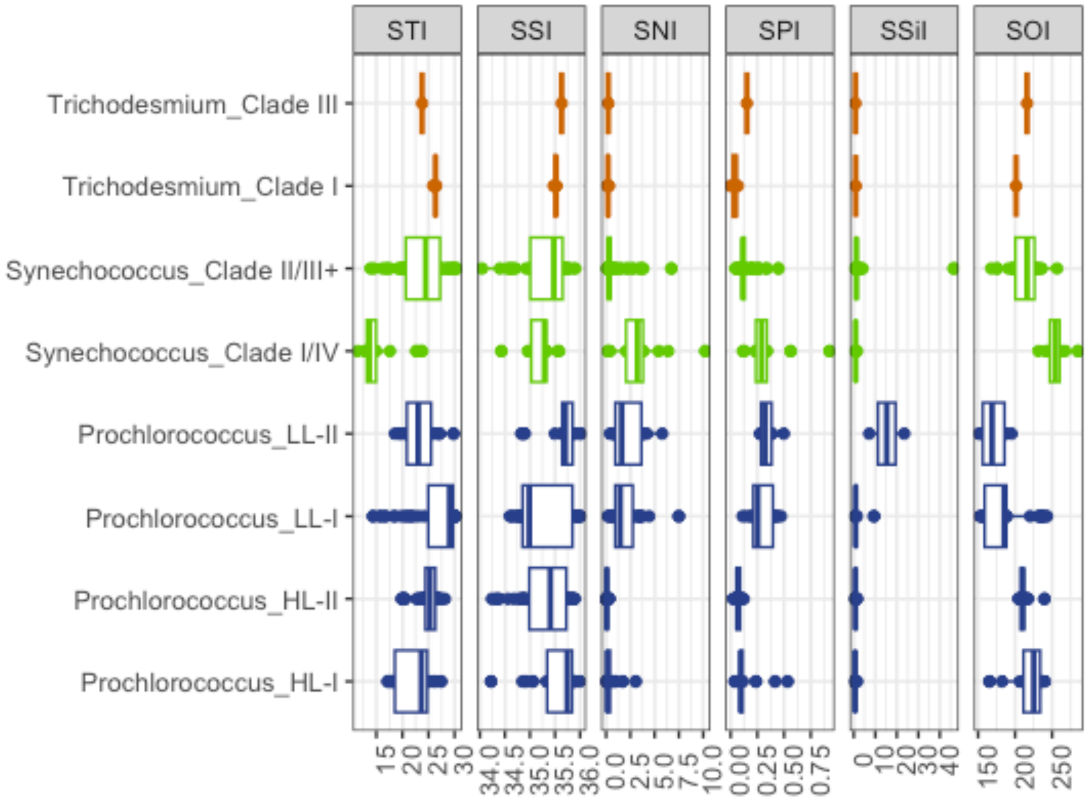

Supplementary Figure 32. Niche differentiation of marine cyanobacterial species and clades resolved using 16S rRNA gene sequences.

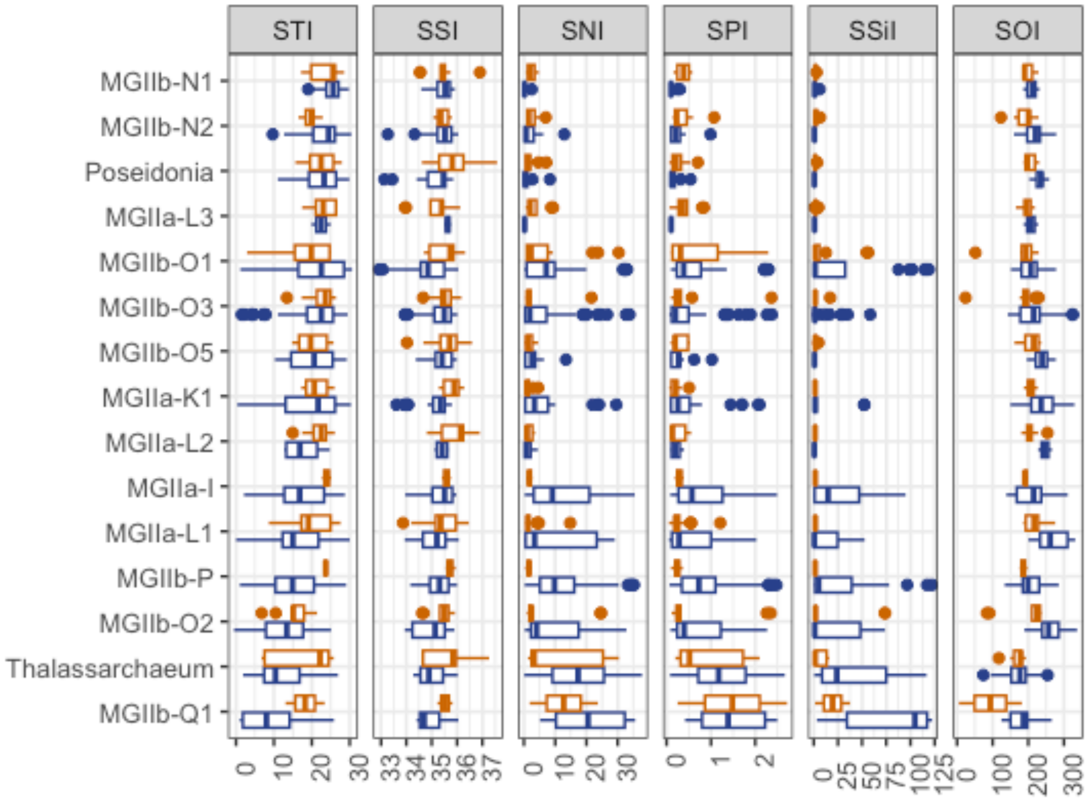

Supplementary Figure 33. Niche differentiation between genera within the Marine Group II Archaea (Poseidoniales). AM (blue) indices derived from the Australian Microbiome archaeal 16S rRNA gene amplicon dataset. TARA (Orange) indices derived from read mapping of TARA Oceans metagenomic data against 204 metagenome assembled genomes and TARA oceans environmental parameters.

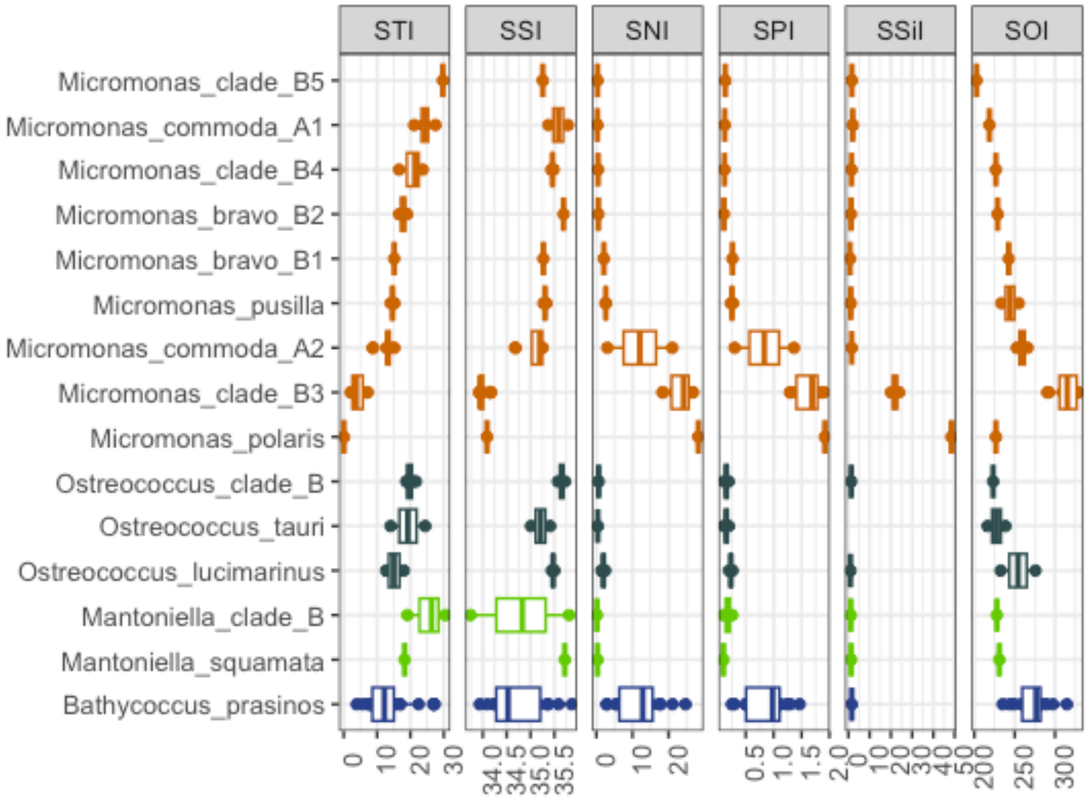

Supplementary Figure 34. Niche differentiation between species and clades in the Order Mamiellales, resolved using chloroplast 16S rRNA gene sequences from the bacterial dataset.

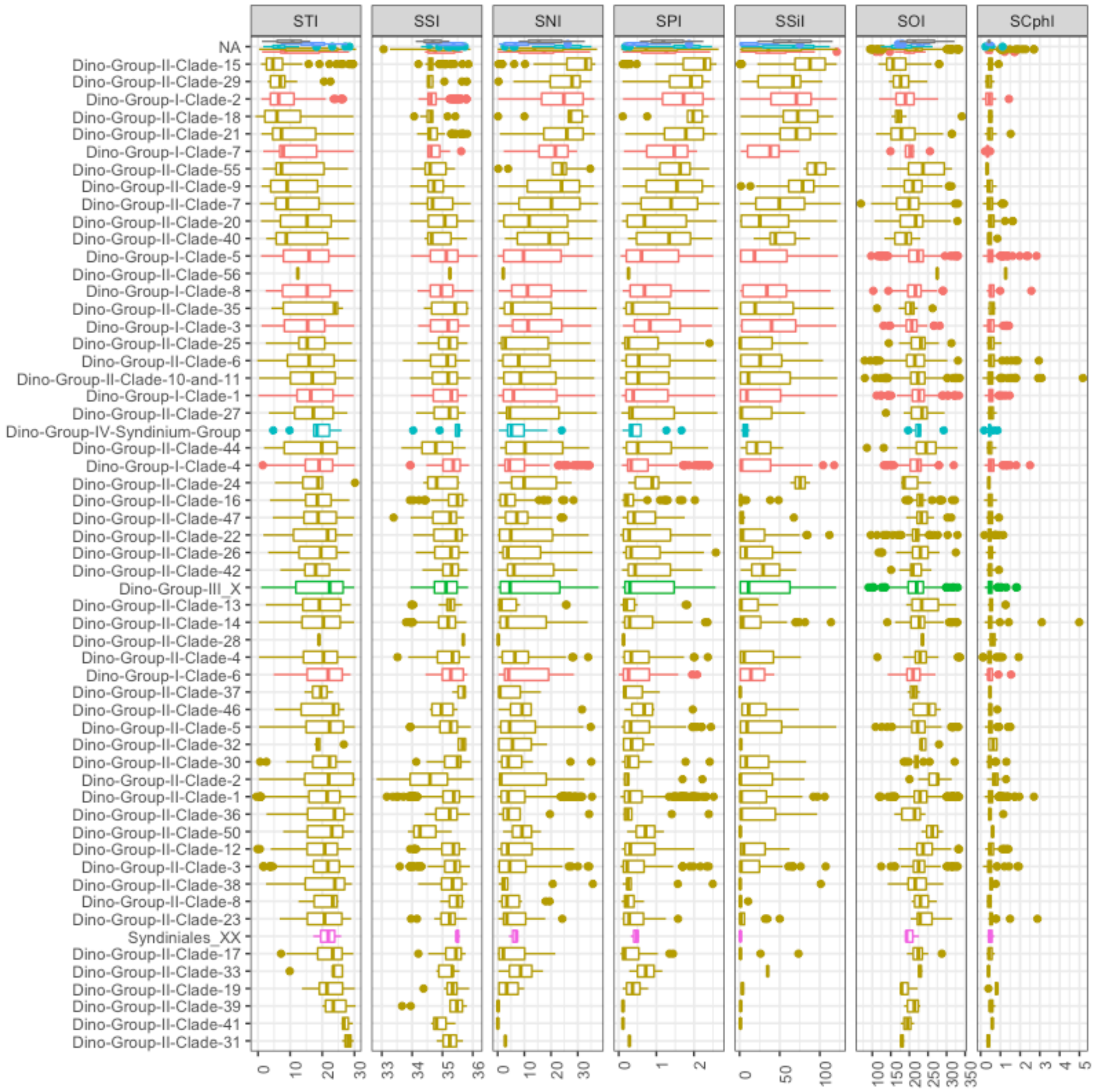

Supplementary Figure 35. Niche differentiation between clades in the Family Syndiniales resolved using 18S rRNA gene sequence data.

Supplementary Table 1: Summary statistics of environmental conditions represented in the dataset.

	Samples with variable measured	Min	1st Quantile	Median	Mean	3rd Quantile	Max
Temperature (°C)	3516	-1.63	8.78	17.28	15.69	21.84	31.84
Salinity (psu)	3450	29.34	34.7	35.33	35.13	35.57	36.39
Nitrate + Nitrite (µmol/L)	3367	0	0.1	1.97	8.85	15.2	39.3
Phosphate (µmol/L)	3376	0	0.11	0.27	0.69	1.09	2.8
Silicate (µmol/L)	3375	0	0.7	1.5	16.43	5.2	139.97
Oxygen (µmol/kg)	2675	9.36	190.6	210.7	212.3	234.45	377.43
Latitude (decimal °)	3664	-66.33	-42.6	-33	-32.61	-27.35	0
Depth (m)	3664	2	10	50	451.4	125	6015.1
Bottom Depth (m)	3533	16	88	1152	2276	4865	5925

Supplementary Table 2: Details of the number of ASVs for which species niche estimates were generated. Total ASVs only includes those with > 100 replicate estimates. Proportion relates to the average proportion of the assemblages these ASVs represent. Mean/ median Std Dev of niche estimates from at least 100 and maximum 1000 repeat estimates.

**Bacteria**	**Species Temperature Index (STI)**	**Species Nitrogen Index (SNI)**	**Species Phosphate Index (SPI)**	**Species Silicate Index (SSiI)**	**Species Salinity Index (SSI)**	**Species Oxygen Index (SOI)**
Total ASVs	16 668	9087	10735	4864	11663	9210
Proportion	97.9	97	94	84.5	94.4	92.7
Median /mean Std. Dev.	0.59 / 0.87 °C	1.17 / 1.70 µmol.l^{-1}	0.07 / 0.11 µmol. l^{-1}	2.66 / 7.0 µmol. l^{-1}	0.08 / 0.13 psu	8.22 / 11.45 µmol.kg^{-1}
**Archaea**						
Total ASVs	2473	1345	1525	754	1416	513
Proportion	95.2	92.4	93.8	89.4	93.8	90.4
Median /mean Std. Dev.	0.59 / 0.89 °C	1.00 / 1.56 µmol.l^{-1}	0.07 / 0.11 µmol.l^{-}	4.57 / 7.57 µmol.l^{-}	0.07 / 0.12 psu	14.30 / 16.72 µmol.kg^{-1}
**Eukaryote**						
Total ASVs	14702	7414	8060	3076	10029	7177
Proportion	93.4	88	89.4	77.6	90.4	87.6
Median /mean Std. Dev.	1.0 / 1.6 °C	1.7 / 2.7 µmol.l^{-1}	0.10 / 0.18 µmol.l^{-1}	7.1 / 12.3 µmol.l^{-1}	0.14 / 0.19 psu	7.1 / 12.2 / µmol.kg^{-1}

Supplementary Table 3: Parameter estimates from linear regression analysis of CTI against *in situ* temperature for subsets of the data.

Station	*n*	Estimate	Std. Error	t value	r^2 Sig	F-Statistic(DF)	p
**Bacteria**							
All sites	3510	0.89	0.004	249.96	0.947	6.248e+04(1,3508)	<2.2e-16
Voyage	1983	0.888	0.004	196.64	0.951	3.867e+04(1,1981)	<2.2e-16
Voyage <10 °C	892	0.705	0.011	64.83	0.825	4203(1,890)	<2.2e-16
Voyage > 10°C & <20°C	503	0.98	0.031	31.69	0.667	1004(1,501)	<2.2e-16
Voyage > 20 °C	542	0.486	0.017	28.27	0.596	799(1,540)	<2.2e-16
MAI	374	0.378	0.011	33.52	0.751	1124(1,372)	<2.2e-16
KAI	67	0.324	0.026	12.59	0.705	158.6(1,65)	<2.2e-16
ROT	190	0.371	0.068	5.42	0.131	29.4(1,188)	1.79E-07
PHB	333	0.767	0.027	27.8	0.699	772.6(1,331)	<2.2e-16
NSI	336	0.542	0.028	19.31	0.526	373(1,334)	<2.2e-16
YON	153	0.13	0.037	3.507	0.069	12.3(1,151)	0.0006
DAR	74	0.241	0.069	3.47	0.131	12.04(1,72)	0.0009
**Archaea**							
**Station**	***n***	**Estimate**	**Std. Error**	**t value**	**r^2 Sig**	**F-Statistic(DF)**	**p**
All sites	2939	0.934	0.003	280.37	0.964	7.86e+04(1,2937)	<2.2e-16
Voyage	1465	0.931	0.004	241.4	0.976	5.829e+04(1,1463)	<2.2e-16
Voyage <10 °C	682	0.696	0.017	39.72	0.698	1578(1,680)	<2.2e-16
Voyage > 10°C & <20°C	365	0.908	0.028	32.924	0.748	1084(1,363)	<2.2e-16
Voyage > 20 °C	418	0.795	0.014	55.95	0.882	3131(1,416)	<2.2e-16
MAI	373	0.219	0.01	21.05	0.543	443(1,371)	<2.2e-16
KAI	67	0.201	0.033	6.125	0.356	37.51(1,65)	5.91E-08
ROT	172	0.389	0.065	5.964	0.168	35.57(1,170)	1.39E-08
PHB	333	0.919	0.026	34.693	0.784	1204(1,331)	<2.2e-16
NSI	335	0.862	0.287	30.07	0.73	904.2(1,333)	<2.2e-16
YON	122	0.274	0.031	8.753	0.385	76.62(1,120)	1.56E-14
DAR	72	0.104	0.062	1.688	0.025	2.85(1,70)	0.096
**Eukaryote**							
**Station**	***n***	**Estimate**	**Std. Error**	**t value**	**r^2 Sig**	**F-Statistic(DF)**	**p**
All sites	2406	0.776	0.005	161.7	0.916	2.615e+04(1,2404)	<2.2e-16
Voyage	1489	0.757	0.006	122.5	0.91	1.5e+04(1,1487)	<2.2e-16
Voyage <10 °C	683	0.663	0.028	24.06	0.459	579(1,681)	<2.2e-16
Voyage > 10°C & <20°C	387	0.644	0.049	13.156	0.308	173.1(1,385)	<2.2e-16
Voyage > 20 °C	419	0.515	0.021	24.7	0.593	610.2(1,417)	<2.2e-16
MAI	203	0.465	0.022	21.5	0.696	462.4(1,201)	<2.2e-16
KAI	67	0.557	0.052	10.76	0.635	115.7(1,65)	4.53E-16
ROT	172	0.29	0.052	5.568	0.149	31(1,170)	9.88E-08
PHB	146	0.799	0.052	15.26	0.615	232.8(1,144)	<2.2e-16
NSI	132	0.601	0.045	13.247	0.571	175.5(1,130)	<2.2e-16
YON	123	0.342	0.038	8.91	0.391	79.39(1,121)	6.32E-15
DAR	74	0.427	0.087	4.295	0.242	24.25(1,72)	5.21E-06

Supplementary Table 4 Details of heatwave activity at Maria Island National Reference Station during the sampling period. Data taken from marineheatwaves.org. The major events of the Austral summers in 2015/16 and 2017/18 and 2018/19 are highlighted in red.

Event No	Date start	Date peak	Date end	Duration	Intensity mean	Intensity max	Intensity cumulative
55	21/4/13	25/4/13	25/4/13	5	1.464	1.64	7.32
56	11/5/13	12/5/13	15/5/13	5	1.572	2.02	7.86
57	21/5/13	24/5/13	5/6/13	16	1.586	1.95	25.37
58	10/7/13	18/7/13	21/7/13	12	0.897	1.04	10.77
59	25/7/13	28/7/13	31/7/13	7	0.936	1.14	6.55
60	14/8/13	17/8/13	18/8/13	5	0.784	0.81	3.92
61	31/8/13	3/9/13	5/9/13	6	1.037	1.26	6.22
62	19/9/13	21/9/13	25/9/13	7	0.98	1.23	6.86
63	28/3/14	5/4/14	22/4/14	26	1.791	2.54	46.56
64	23/5/14	27/5/14	7/6/14	16	1.5	2.31	24
65	27/6/14	1/7/14	11/7/14	15	0.899	1.18	13.49
66	12/8/14	20/8/14	29/8/14	18	0.859	1	15.47
67	14/4/15	16/4/15	19/4/15	6	1.535	1.61	9.21
68	6/9/15	24/11/15	27/11/15	83	1.394	2.2	115.66
69	4/12/15	8/2/16	9/5/16	158	2.428	3.72	383.64
70	1/6/16	2/6/16	6/6/16	6	1.598	1.71	9.59
71	14/6/16	15/6/16	23/6/16	10	1.369	1.7	13.69
72	1/7/16	3/7/16	6/7/16	6	0.963	1.2	5.78
73	20/9/16	20/9/16	5/10/16	16	1.035	1.39	16.56
74	5/1/17	9/1/17	15/2/17	42	1.835	3.19	77.09
75	16/5/17	19/5/17	24/5/17	9	1.906	2.31	17.15
76	7/6/17	13/6/17	27/6/17	21	1.504	1.97	31.59
77	4/8/17	9/8/17	10/8/17	7	0.95	1.11	6.65
78	27/10/17	29/10/17	1/11/17	6	1.337	1.45	8.02
79	16/11/17	30/11/17	12/1/18	58	2.343	3.79	135.87
80	18/1/18	28/1/18	18/2/18	32	2.116	2.92	67.71
81	28/2/18	16/3/18	19/3/18	20	1.696	1.98	33.92
82	2/5/18	5/5/18	21/5/18	20	1.704	2.31	34.07
83	27/5/18	30/5/18	11/6/18	16	1.324	1.59	21.19
84	14/7/18	15/7/18	18/7/18	5	1.234	1.43	6.17
85	27/11/18	30/11/18	1/12/18	5	1.628	1.76	8.14
86	22/12/18	3/1/19	11/2/19	52	2.049	2.92	106.52
87	7/6/19	11/6/19	13/6/19	7	1.197	1.42	8.38
88	8/10/19	10/10/19	14/10/19	7	1.147	1.5	8.03

Supplementary Table 5: Parameter estimates from linear regression and Pearson correlation analysis of community indices against in situ variables at the MAI

**Bacteria**	Location	*n*	Estimate	Std Error	t value	r^2 Sig	F-Statistic(DF)	p	Pearsons *r*	Pearson p
CTI ~ Temperature	All	3510	0.89	0.004	249.96	0.947	6.248e+04(1,3508)	<2.2e-16	0.97	<2.2e-16
CTI ~ Temperature	MAI	374	0.378	0.011	33.52	0.751	1124(1,372)	<2.2e-16	0.87	<2.2e-16
CSI ~ salinity†	All	3282	0.722	0.007	99.84	0.752	9968(1,3280)	<2.2e-16	0.87	<2.2e-16
CSI ~ salinity†	MAI	380	0.379	0.012	31.18	0.72	971.9(1,377)	<2.2e-16	0.85	<2.2e-16
CNI ~ nitrite+nitrate	All	3340	0.887	0.003	256.86	0.952	6.598e+04(1,3338)	<2.2e-16	0.98	<2.2e-16
CNI ~ nitrite+nitrate	MAI	419	0.36	0.019	18.21	0.44	331.6(1,417)	<2.2e-16	0.67	<2.2e-16
CPI ~ PO4	All	3349	0.893	0.004	235.12	0.94	5.528e+04(1,3347)	<2.2e-16	0.97	<2.2e-16
CPI ~ PO4	MAI	419	0.303	0.019	15.92	0.38	253.4(1,417)	<2.2e-16	0.61	<2.2e-16
CSiI ~ silicate	All	3348	0.86	0.007	122.3	0.82	1.495e+04(1,3346)	<2.2e-16	0.9	<2.2e-16
CSiI ~ silicate	MAI	419	0.53	0.096	5.51	0.07	30.33(1,417)	6.37E-08	0.26	6.37E-08
COI ~ oxygen	All		0.55	0.011	49.98	0.48	2499(1,2653)	<2.2e-16	0.7	<2.2e-16
COI ~ oxygen	MAI	354	0.085	0.008	11.09	0.26	122.9(1,352)	<2.2e-16	0.51	<2.2e-16
**Archaea**										
CTI ~ Temperature	All	2939	0.934	0.003	280.37	0.964	7.86e+04(1,2937)	<2.2e-16	0.74	<2.2e-16
CTI ~ Temperature	MAI	373	0.219	0.01	21.05	0.543	443(1,371)	<2.2e-16	0.74	<2.2e-16
CSI ~ salinity†	All	2907	0.805	0.007	122.7	0.838	1.506+e04(1,2905)	<2.2e-16	0.92	<2.2e-16
CSI ~ salinity†	MAI	378	0.283	0.013	21.73	0.556	472.2(1,376)	<2.2e-16	0.75	<2.2e-16
CNI ~ nitrite+nitrate	All	3012	0.915	0.004	251.13	0.954	6.307e+04(1,3010)	<2.2e-16	0.98	<2.2e-16
CNI ~ nitrite+nitrate	MAI	418	0.297	0.017	17.07	0.411	291.4(1,416)	<2.2e-16	0.64	<2.2e-16
CPI ~ PO4	All	3021	0.916	0.004	227.78	0.945	5.189e+04(1,3019)	<2.2e-16	0.97	<2.2e-16
CPI ~ PO4	MAI	418	0.238	0.015	15.43	0.363	238.2(1,416)	<2.2e-16	0.6	<2.2e-16
CSiI ~ silicate	All	3020	0.888	0.008	117.12	0.82	1.372e+04(1,3018)	<2.2e-16	0.91	<2.2e-16
CSiI ~ silicate	MAI	418	0.235	0.009	24.1	0.582	580.7(1,416)	<2.2e-16	0.76	<2.2e-16
COI ~ oxygen	All	2450	0.587	0.015	39.26	0.381	1542(1,2498)	<2.2e-16	0.62	<2.2e-16
COI ~ oxygen	MAI	353	0.09	0.005	7.456	0.134	55.59(1,351)	7.04E-13	0.37	7.05E-13
**Eukaryote**										
CTI ~ Temperature	All	2406	0.776	0.005	161.7	0.916	2.615e+04(1,2404)	<2.2e-16	0.95	<2.2e-16
CTI ~ Temperature	MAI	203	0.465	0.022	21.5	0.696	462.4(1,201)	<2.2e-16	0.84	<2.2e-16
CSI ~ salinity†	All	2348	0.584	0.007	83.06	0.746	6898(1,2346)	<2.2e-16	0.86	<2.2e-16

CSI ~ salinity†	MAI	212	0.409	0.03	13.74	0.471	188.8(1,210)	<2.2e-16	0.68	<2.2e-16
CNI ~ nitrite+nitrate	All	2424	0.771	0.004	177.06	0.928	3.135e+04(1, 2422)	<2.2e-16	0.96	<2.2e-16
CNI ~ nitrite+nitrate	MAI	228	0.518	0.047	11.12	0.351	123.6(1,226)	<2.2e-16	0.59	<2.2e-16
CPI ~ PO4	All	2428	0.789	0.005	164.74	0.918	2.714e+04(1, 2426)	<2.2e-16	0.96	<2.2e-16
CPI ~ PO4	MAI	228	0.471	0.041	11.49	0.366	132.1(1,226)	<2.2e-16	0.61	<2.2e-16
CSiI ~ silicate	All	2428	0.718	0.008	89.4	0.767	7992(1,2462)	<2.2e-16	0.88	<2.2e-16
CSiI ~ silicate	MAI	228	1	0.177	5.637	0.119	31.78(1,226)	5.12E-08	0.35	5.12E-08
COI ~ oxygen	All	2070	0.44	0.011	39.66	0.412	1573(1,2068)	<2.2e-16	0.66	<2.2e-16
COI ~ oxygen	MAI	183	0.291	0.02	14.31	0.528	204.9(1,181)	<2.2e-16	0.73	<2.2e-16

Supplementary Table 6: Statistical tests on bacterial diversity and occurrence rate, associated with Figure 6. Statistically significant results in bold.

A) Parameters from T-tests comparing Bacterial Inverse Simpsons Index at Maria Island during MHW and non-MHW months				
	**t**	**p**	**mean during MHW**	**mean during Non-MHW**
Bacteria All	t(236) = 1.6	0.1	127.6	118.9
Surface	t(95) = 0.6	0.5	119.5	114.3
Mid	t(78) = -0.01	0.1	128.1	128.2
**Deep**	**t(80) = 2.13**	**0.03**	**135.7**	**115.9**
January	t(20) = 0.38	0.7	77.3	72.6
February	t(34) = -0.77	0.4	113.2	120.7
**March**	**t(55) = 2.83**	**0.006**	**140.3**	**110**
**April**	**t(30) = 5.76**	**2.80E-06**	**168.9**	**97.3**
May	t(13) = -0.1	0.9	134.6	135.8
June	t(11)= 1.39	0.2	172.8	152.4
July	t(6) = -0.7	0.5	168.5	188.2
**August**	**t(13) = 3.15**	**0.008**	**195.3**	**166.8**
September	t(21) = 1.1	0.2	104.5	87.9
October	t(37) = 0.34	0.7	83	80.2
**November**	**t(18) = 4.9**	**0.0001**	**94.2**	**49.5**
**December**	**t(25) = 2.68**	**0.01**	**74.9**	**59.1**
B) Parameters from T-tests comparing Bacterial Shannon diversity Index at Maria Island during MHW and non-MHW months. Statistically significant reults in bold.				
	**t**	**p**	**mean during MHW**	**mean during Non-MHW**
**Bacteria All**	**t(340) = 3.24**	**0.001**	**6.38**	**6.23**
Surface	t(123) = 1.02	0.3	6.23	6.14
Mid	t(114) = 1.3	0.2	6.4	6.3
**Deep**	**t(116) = 4.0**	**0.0001**	**6.54**	**6.27**
January	t(23) = 1.15	0.2	5.95	5.76
February	t(14) = -0.1	0.9	6.39	9.39
**March**	**t(43) = 2.26**	**0.03**	**6.55**	**6.25**
**April**	**t(33) = 6.00**	**9.38E-07**	**6.72**	**6.37**

May	t(32) = -0.2	0.9	6.54	6.54
June	t(15) = 0.67	0.5	6.65	6.6
July	t(11) = -0.57	0.5	6.64	6.69
**August**	**t(16) = 2.10**	**0.05**	**6.7**	**6.6**
**September**	**t(33) = 3.27**	**0.002**	**6.32**	**5.82**
October	t(37) = -1.1	0.3	5.62	5.73
**November**	**t(27) = 4.2**	**0.0003**	**6.13**	**5.39**
**December**	**t(24) = 3.7**	**0.001**	**6.01**	**5.58**

C) Parameters from T-tests comparing the number of uncommon Bacterial taxa at Maria Island during MHW and non-MHW months. Statistically significant results in bold.

	**t**	**p**	**mean during MHW**	**mean during Non-MHW**
**Bacteria All**	**t(152) = 3.70**	**0.0003**	**27.05**	**19.55**
**Surface**	**t(50) = 2.68**	**0.01**	**30**	**20.33**
**Mid**	**t(54) = 2.11**	**0.04**	**23.34**	**17.8**
Deep	t(48) = 1.81	0.08	27.8	20.42
January	t(13) = 0.18	0.8	33.5	32.25
February	t(5) = 1.73	0.1	49	26.22
**March**	**t(25) = 3.96**	**0.0006**	**41.56**	**20.26**
**April**	**t(19) = 3.69**	**0.002**	**29.68**	**12**
**May**	**t(29) = 5.25**	**1.28E-05**	**16.14**	**9.33**
**June**	**t(17) = 4.99**	**0.0001**	**15**	**9.69**
**July**	**t(33) = 2.81**	**0.008**	**22.58**	**20.42**
**August**	**t(41) = -6.30**	**1.56E-07**	**4.67**	**15.18**
**September**	**t(20) = -2.93**	**0.008**	**9.5**	**23.53**
**October**	**t(30) = 3.31**	**0.002**	**35.33**	**26.77**
November	t(10) = 2.14	0.06	41.22	26.89
December	t(21) = -0.52	0.6	23.89	26.38

Supplementary Table 7: Statistical tests on archaeal diversity and occurrence rate associated with Supplementary Figure 26. Statistically significant results in bold.

A) Parameters from T-tests comparing Archaeal Inverse Simpsons Index at Maria Island during MHW and non-MHW months

	**t**	**p**	**mean during MHW**	**mean during Non-MHW**
Archaea All	t(255) = -2.18	0.03	7.8	8.64
Surface	t(77) = -0.84	0.4	7.96	8.6
**Mid**	**t(96) = -2.39**	**0.02**	**7.41**	**8.93**
Deep	t(95) = -0.85	0.4	7.97	8.45
January	t(17) = -1.26	0.3	4.32	5.31
February	t(11) = -0.67	0.5	6.16	6.96
**March**	**t(40) = 3.02**	**0.004**	**9.81**	**7.32**
**April**	**t(36) = 1.16**	**0.02**	**9.15**	**7.97**
**May**	**t(9) = 3.05**	**0.006**	**8.42**	**6.56**
June	t(11) = -1.25	0.2	7	7.83
July	t(7) = -0.66	0.5	10.38	11.32
August	t(8) = -0.76	0.5	13.94	14.92

September	t(28) = -0.05	0.9	9.52	9.58
**October**	**t(37) = -5.70**	**1.63E-06**	**5.09**	**8.12**
November	t(22) = -0.85	0.4	5.29	5.75
December	t(23) = -1.66	0.1	3.88	5.12
B) Parameters from T-tests comparing Archaeal Inverse Simpsons Index at Maria Island during MHW and non-MHW months				
	**t**	**p**	**mean during MHW**	**mean during Non-MHW**
Archaea All	t(219) = -0.69	0.5	3.24	3.29
Surface	t(77) = -1.34	0.2	3.07	3.26
Mid	t(72) = -1.05	0.3	3.18	3.31
Deep	t(74) = 1.34	0.2	3.47	3.31
January	t(16) = -0.62	0.5	2.41	2.57
February	t(13) = 0.39	0.7	3.16	3.04
**March**	**t(53) = 3.67**	**0.005**	**3.69**	**3.15**
April	t(39) = 1.01	0.3	3.56	3.37
May	t(15) = 0.34	0.7	3.28	3.24
June	t(15) = 0.26	0.8	3.51	3.47
**July**	**t(21) = -4.75**	**0.0001**	**3.34**	**3.86**
August	t(17) = 0.61	0.5	4.1	4.04
September	t(32) = 0.71	0.5	3.64	3.53
**October**	**t(35) = -9.13**	**8.23E-11**	**2.37**	**3.05**
November	t(13) = 1.36	0.2	2.86	2.56
December	t(22) = -0.26	0.8	2.29	2.35
C) Parameters from T-tests comparing the number of uncommon Archaeal taxa at Maria Island during MHW and non-MHW months				
	**t**	**p**	**meanHW**	**meanNHW**
Archaea All	t(270) = -0.95	0.3	13.1	15.43
**Surface**	**t(136) = -1.98**	**0.05**	**9.16**	**15.67**
Mid	t(107) = -0.63	0.5	14.68	17.59
Deep	t(56) = 0.45	0.6		
January	t(7) = 1.02	0.3	22.63	2.64
February	t(27) = -0.86	0.4	8.5	11.66
**March**	**t(35) = -2.03**	**0.05**	**7.17**	**14.58**
April	t(31) = -0.3	0.8	17.17	19.82
May	t(24) = -2.00	0.06	6.57	19.46
**June**	**t(36) = -2.14**	**0.04**	**5.67**	**15.8**
July	t(13) = 0.18	0.9	15	13.79
August	t(6) = 0.66	0.5	28	20.29
September	t(12) = 1.00	0.3	14	7.6
October	t(26) = -0.92	0.4	12.33	21.23
November	t(32) = 0.84	0.4	14.22	10.07
December	t(14) = -1.05	0.3	4.89	16.2

Supplementary Table 8: Statistical tests on eukaryotic diversity and occurrence rate, associated with Supplementary Figure 27. Statistically significant results in bold.

A) Parameters from T-tests comparing Eukaryote Inverse Simpsons Index at Maria Island during MHW and non-MHW months				
	**t**	**p**	**mean during MHW**	**mean during Non-MHW**
Eukaryote All	t(175) = -1.71	0.09	36.73	42.95
Surface	t(85) = -2.20	0.03	27.21	37.07
Mid	t(55) = -1.10	0.3	38.29	46.07
Deep	t(50) = -0.14	0.9	45.97	47
January	t(6) = -0.8	0.4	24.81	35.27
February	t(22) = -1.2	0.2	38.73	55.9
March	t(36) = 1.51	0.1	46.77	34.53
**April**	**t(26) = -4.98**	**3.62E-05**	**11.72**	**41.2**
May	NA	NA	NA	NA
**June**	**t(30) = -3.04**	**0.005**	**40.03**	**63.2**
July	NA	NA	NA	NA
August	NA	NA	NA	NA
September	t(12) = 0.34	0.74	24.48	22.3
October	t(16) = 7.19	0.4	28.86	24
November	t(21) = 0.20	0.7	29.6	25.96
**December**	**t(11) = 2.15**	**0.05**	**62.54**	**22.05**
B) Parameters from T-tests comparing Eukaryote Shannons Index at Maria Island during MHW and non-MHW months. Statistically significant results in bold				
	**t**	**p**	**mean during MHW**	**mean during Non-MHW**
Eukaryote All	t(195) = -0.99	0.3	4.86	4.95
Surface	t(88) = -0.99	0.3	4.6	4.76
Mid	t(47) = -1.04	0.3	4.82	5.02
Deep	t(63) = 0.42	0.6	5.17	5.12
January	t(9) = -1.30	0.2	4.57	4.94
February	t(18) = -1.28	0.2	4.81	5.19
**March**	**t(22) = 2.13**	**0.04**	**5.31**	**4.67**
**April**	**t(7) = -4.99**	**0.001**	**3.71**	**5.17**
May	NA	NA	NA	NA
**June**	**t(12) = -1.95**	**0.07**	**5.24**	**5.5**
July	NA	NA	NA	NA
August	NA	NA	NA	NA
**September**	**t(17) = 1.93**	**0.07**	**4.75**	**4.27**
October	t(13) = 0.35	0.7	4.5	4.42
November	t(16) = 1.37	0.2	4.61	4.1
**December**	**f(9) = 2.79**	**0.02**	**5.23**	**4.12**
C) Parameters from T-tests comparing the number of uncommon Eukaryote taxa at Maria Island during MHW and non-MHW months. Statistically significant results in bold.				
	**t**	**p**	**mean during MHW**	**mean during Non-MHW**
**Eukaryote All**	**t(122) = 3.77**	**0.0003**	**35.99**	**23.55**
**Surface**	**t(47) = 2.11**	**0.04**	**33.23**	**23.04**
**Mid**	**t(38) = 2.43**	**0.02**	**30.79**	**19.12**
**Deep**	**t(37) = 2.39**	**0.02**	**43.26**	**27.05**
January	t(6) = 0.67	0.5	35.63	29

February	t(7) = 0.62	0.6	43.83	33.92
March	t(16) = -1.66	0.1	33.5	45.92
**April**	**t(6) = 7.78**	**0.0004**	**90**	**16.41**
May	NA	NA	NA	NA
June	t(2) = 1.79	0.2	18.67	7.83
**July**	**t(20) = -2.34**	**0.03**	**6.33**	**18.12**
August	NA	NA	NA	NA
**September**	**t(9) = -2.32**	**0.04**	**18.83**	**39.11**
**October**	**t(9) = -4.30**	**0.002**	**23.17**	**38.17**
**November**	**t(13) = 1.79**	**0.05**	**53.56**	**37.5**
**December**	**t(5) = -3.08**	**0.02**	**28**	**47.75**

Supplementary Table 9. Comparison of community indices generated herein with those generated through the Australian Microbiome using identical code but alternative bioinformatic protocols. KD = Kernel density approach to identifying STI, mean = mean approach to identifying STI. #1 are the indices using the bioinformatic protocols described in the methods, #2 are the indices using the bioinformatic protocols of the Australian Microbiome described here https://github.com/AusMicrobiome/amplicon/tree/master/docs

Linear model parameters	Estimate	Std. Error	t value	R2	F-statistic(DF)	p
**Community Temperature index**						
Bacteria_KD1~mean1	0.974	0.0005	1639.57	0.999	2.688e+06(1,3639)	<2.2e-16
Bacteria_KD2~mean2	0.978	0.0007	1436.27	0.999	2.063e+06(1,3182)	<2.2e-16
Bacteria_KD1~KD2	1.006	0.0007	1427.81	0.998	2.039e+06(1,3182)	<2.2e-16
Bacteria_mean1~mean2	1.001	0.0007	1345.52	0.998	1.81e+06(1,3182)	<2.2e-16
Bacteria_KD1~mean2	0.971	0.001	1114	0.997	1.241e+06(1,3182)	<2.2e-16
Bacteria_KD2~mean1	0.98	0.001	1032.79	0.997	1.067e+06(1,3182)	<2.2e-16
Archaea-KD1~mean1	0.979	0.0008	1211.02	0.998	1.467e+06(1,3104)	<2.2e-16
Archaea_KD2~mean2	0.984	0.0005	1783.96	0.999	3.183e+06(1,3087)	<2.2e-16
Archaea_KD1~KD2	0.998	0.002	594.869	0.992	3.539e+05(1,3034)	<2.2e-16
Archaea_mean1~mean2	0.994	0.002	635	0.993	4.032e+05(1,3034)	<2.2e-16
Archaea_KD1~mean2	0.978	0.002	553.68	0.99	3.066e+05(1,3034)	<2.2e-16
Archaea_KD2~mean1	0.979	0.002	608.16	0.992	3.699e+05(1,3034)	<2.2e-16
Eukaryote_KD1_mean1	1.044	0.001	722.93	0.995	5.226e+05(1,2542)	<2.2e-16
Eukaryote_KD2_mean2	1.04	0.001	759.6	0.996	5.77e+05(1,2617)	<2.2e-16
Eukaryote_KD1~KD2	0.995	0.002	652.537	0.994	4.258e+05(1,2436)	<2.2e-16
Eukaryote_mean1~mean2	1.002	0.001	656.24	0.994	4.307e+05(1,2436)	<2.2e-16
Eukaryote_KD1~mean2	1.036	0.002	511.2	0.991	2.613e+05(1,2435)	<2.2e-16
Eukaryote_KD2~mean1	1.0439	0.002	521.01	0.991	2.715e+05(1,2436)	<2.2e-16
**Nitrite-Nitrate**						
Bacteria-KD1-KD2	0.984	0.002	565.536	0.99	3.198e+05(1,3182)	<2.2e-16
Bacteria-mean1-mean2	0.977	0.001	636.55	0.992	4.052e+05(1,3812)	<2.2e-16
Archaea-KD1-KD2	0.986	0.001	592.26	0.991	3.508e=0591,3034)	<2.2e-16
Archaea-mean1-mean2	0.976	0.002	586.07	0.991	3.435e+05(1,3034)	<2.2e-16

Eukaryote-KD1-KD2	0.944	0.002	418.89	0.986	1.755e+05(1,2436)	<2.2e-16
Eukaryote-mean1-mean2	0.935	0.002	417.42	0.986	1.742e+05(1,2435)	<2.2e-16
**Phosphate**						
Bacteria-KD1-KD2	0.969	0.002	544.93	0.989	2.97e+05(1,3182)	<2.2e-16
Bacteria-mean1-mean2	0.944	0.001	680.36	0.993	4.629e+05(1,3182)	<2.2e-16
Archaea-KD1-KD2	0.979	0.001	591.84	0.991	3.503e+05(1,3034)	<2.2e-16
Archaea-mean1-mean2	0.951	0.002	600.55	0.992	3.607e+05(1,3034)	<2.2e-16
Eukaryote-KD1-KD2	0.929	0.002	399.18	0.985	1.593e+05(1,2436)	<2.2e-16
Eukaryote-mean1-mean2	0.913	0.002	391.57	0.984	1.533e+05(1,2436)	<2.2e-16
**Salinity**						
Bacteria-KD1-KD2	0.938	0.008	117.53	0.813	1.381e+04(1,3182)	<2.2e-16
Bacteria-mean1-mean2	0.843	0.015	55.53	0.492	3084(1,3182)	<2.2e-16
Archaea-KD1-KD2	0.889	0.007	117.76	0.82	1.387e+04(1,3034)	<2.2e-16
Archaea-mean1-mean2	0.736	0.013	55.55	0.504	3086(1,3034)	<2.2e-16
Eukaryote-KD1-KD2	0.8	0.012	62.27	0.614	3877(1,2436)	<2.2e-16
Eukaryote-mean1-mean2	0.574	0.03	37.74	0.234	769.5(1,2436)	<2.2e-16
**Silicate**						
Bacteria-KD1-KD2	1.01	0.001	605.184	0.991	3.662e+05(1,3182)	<2.2e-16
Bacteria-mean1-mean2	0.971	0.002	509.83	0.988	2.599e+05(1,3182)	<2.2e-16
Archaea-KD1-KD2	1.01	0.002	473.36	0.987	2.241e+05(1,3034)	<2.2e-16
Archaea-mean1-mean2	0.944	0.004	265.4	0.959	7.044e+04(1,3034)	<2.2e-16
Eukaryote-KD1-KD2	1.04	0.004	225.21	0.954	5.072e+04(1,2436)	<2.2e-16
Eukaryote-mean1-mean2	1.01	0.004	228.07	0.955	5.201e+04(1,2436)	<2.2e-16
**Oxygen**						
Bacteria-KD1-KD2	0.95	0.003	331.2	0.972	1.097e+05(1,3182)	<2.2e-16
Bacteria-mean1-mean2	0.973	0.004	248.9	0.95	6.197e+04(1,3812)	<2.2e-16
Archaea-KD1-KD2	0.792	0.003	238.58	0.949	5.692e+04(1,3034)	<2.2e-16
Archaea-mean1-mean2	0.924	0.004	253.57	0.955	6.43e+04(1,3034)	<2.2e-16
Eukaryote-KD1-KD2	1	0.005	207.47	0.946	4.304e+04(1,2435)	<2.2e-16
Eukaryote-mean1-mean2	0.965	0.004	237.43	0.959	5.637e+04(1,2436)	<2.2e-16

Supplementary Table 10. KD model output parameters for ASVs displayed in Supplementary Figure 31 based on the full 1000 repeat estimations of random subsets from temperature bins. STI-KD = Species temperature index estimated by Kernal density approach; STI-MEAN = species temperature index estimated by mean temperature of the 4 samples in which the ASV displayed the greatest relative abundance; Tmin = temperature minimum, (1/4 KD peak height left); Tmax = temperature maximum (1/4 peak height right); Trange = temperature range (Tmin-Tmax); SD = standard deviation.

ASVid	(A,B) Bc1000005	(C,D) Bc1000017	(E,F) Bc1000071
Phylum	Cyanobacteria	Cyanobacteria	Proteobacteria
Class	Cyanobacteriia	Cyanobacteriia	Alphaproteobacteria
Order	Synechococcales	Synechococcales	SAR11_clade
Family	Cyanobiaceae	Cyanobiaceae	Sar11_Clade_I
Genus	Prochlorococcus MIT9313	Prochlorococcus MIT9313	SAR11_Clade_Ia
STI-KD (SD)	25.12 (0.15)	18.02 (0.23)	23.89 (0.21)
STI-MEAN (SD)	25.87 (0.56)	18.17 (0.42)	24.42 (0.80)
Tmin (SD)	18.73 (0.18)	11.85 (0.30)	14.83 (0.26)
Tmax (SD)	30.97 (0.13)	24.36 (0.32)	31.48 (0.16)
Trange (SD)	12.25 (0.22)	12.51 (0.51)	16.66 (0.34)
ASVid	(G,H) Bc1001410	(I,J) Bc1003019	(K,L) Bc1001974
Phylum	Proteobacteria	Proteobacteria	Bacteroidota
Class	Alphaproteobacteria	Alphaproteobacteria	Bacteroidiia
Order	SAR11_clade	Rhodospirlialles	Flavobacteriales
Family	Sar11_Clade_I	AEGEAN-169_marine_group	Flavobacteriaceae
Genus	SAR11_Clade_Ia	AEGEAN-169_marine_group	NS2b_marine_group
STI-KD (SD)	13.18 (0.18)	14.41 (0.18)	12.38 (0.13)
STI-MEAN (SD)	14.09 (0.55)	14.09 (0.55)	12.26 (0.27)
Tmin (SD)	6.29 (0.31)	8.89 (0.25)	9.47 (0.19)
Tmax (SD)	20.01 (0.29)	19.89 (0.22)	16.01 (0.31)
Trange (SD)	13.72 (0.45)	11.00 (0.37)	6.54 (0.40)
