# Supplementary material for: A marine heatwave drives significant shifts in pelagic microbiology
Source: Commun Biol. 2024 Jan 24;7:125. doi: 10.1038/s42003-023-05702-4 (PMC10808424; doi:10.1038/s42003-023-05702-4)
Supplement: Supplementary file 3 — Description of Additional Supplementary Files [file 42003_2023_5702_MOESM3_ESM.pdf]

Description of Additional Supplementary Files

File name: Supplementary Data 1
Description: Supplementary Data 1 provides metadata for samples used in this study including geographic location (latitude/longitude), location descriptors, depths, collection data, environmental variables measured coincidently and 16S/18S rRNA sequencing and quality control statistics.

File name: Supplementary Data 2
Description: Supplementary Data 2 provides a comparison of the species temperature index for common Southern Hemisphere phytoplankton generated using molecular data (this study) and by continuous plankton recorder and miroscopy (Ajani, P. A., Davies, C. H., Eriksen, R. S. & Richardson, A. J. Global warming impacts micro-phytoplankton at a long-term Pacific Ocean coastal station. *Front. Mar. Sci*. **7**, (2020) DOI=10.3389/fmars.2020.576011)
