# Supplementary material for: A marine heatwave drives significant shifts in pelagic microbiology
Source: Commun Biol. 2024 Jan 24;7:125. doi: 10.1038/s42003-023-05702-4 (PMC10808424; doi:10.1038/s42003-023-05702-4)
Supplement: Supplementary file 5 — Reporting Summary [file 42003_2023_5702_MOESM5_ESM.pdf]

nature portfolio

Corresponding author(s): mark vincent brown

Last updated by author(s): Dec 1, 2023

Reporting Summary

Nature Portfolio wishes to improve the reproducibility of the work that we publish. This form provides structure for consistency and transparency in reporting. For further information on Nature Portfolio policies, see our Editorial Policies and the Editorial Policy Checklist.

Statistics

For all statistical analyses, confirm that the following items are present in the figure legend, table legend, main text, or Methods section.

n/a	Confirmed	
☐	☒	The exact sample size (*n*) for each experimental group/condition, given as a discrete number and unit of measurement
☐	☒	A statement on whether measurements were taken from distinct samples or whether the same sample was measured repeatedly
☐	☒	The statistical test(s) used AND whether they are one- or two-sided<br>*Only common tests should be described solely by name; describe more complex techniques in the Methods section.*
☐	☒	A description of all covariates tested
☐	☒	A description of any assumptions or corrections, such as tests of normality and adjustment for multiple comparisons
☐	☒	A full description of the statistical parameters including central tendency (e.g. means) or other basic estimates (e.g. regression coefficient) AND variation (e.g. standard deviation) or associated estimates of uncertainty (e.g. confidence intervals)
☒	☐	For null hypothesis testing, the test statistic (e.g. *F*, *t*, *r*) with confidence intervals, effect sizes, degrees of freedom and *P* value noted<br>*Give P values as exact values whenever suitable.*
☒	☐	For Bayesian analysis, information on the choice of priors and Markov chain Monte Carlo settings
☒	☐	For hierarchical and complex designs, identification of the appropriate level for tests and full reporting of outcomes
☐	☒	Estimates of effect sizes (e.g. Cohen's *d*, Pearson's *r*), indicating how they were calculated

Our web collection on statistics for biologists contains articles on many of the points above.

Software and code

Policy information about availability of computer code

Data collection	No software was used
Data analysis	R version 3.6.0<br>R Studio Version 1.2.5042<br>DADA2 V1.16<br>USEARCH V11<br>UMAP 0.5<br>Primer V7<br>Custom Code at github.com/AusMicrobiome/microbial_ocean_atlas

For manuscripts utilizing custom algorithms or software that are central to the research but not yet described in published literature, software must be made available to editors and reviewers. We strongly encourage code deposition in a community repository (e.g. GitHub). See the Nature Portfolio guidelines for submitting code & software for further information.

Data

Policy information about availability of data

All manuscripts must include a data availability statement. This statement should provide the following information, where applicable:

- Accession codes, unique identifiers, or web links for publicly available datasets
- A description of any restrictions on data availability
- For clinical datasets or third party data, please ensure that the statement adheres to our policy

Sequence data from the Australian Microbiome is available under NCBI Bioproject number PRJNA385736. All environmental metadata including physical (temperature, salinity, oxygen), biological (chlorophyll) and nutrient parameter are available via the Australian Ocean Data Network Portal (https://portal.aodn.org.au/). In situ temperature, salinity, oxygen, turbidity, chlorophyll Water Quality Meter data for 20m and 85m at Maria Island (https://portal.aodn.org.au/search?uuid=8964658c-6ee1-4015-9bae-2937dfcc6ab9). Near real-time meteorology and sea surface temperature at Maria Island (https://portal.aodn.org.au/search?uuid=f3910f5c-c568-4af0-b773-13c0e57ada6b). Discrete depth temperature, salinity, carbon, nitrate, phosphate, silicate, oxygen, chlorophyll for National Reference Stations (https://portal.aodn.org.au/search?uuid=b442c3e8-3d30-48ad-b144-680afd848167)

Research involving human participants, their data, or biological material

Policy information about studies with human participants or human data. See also policy information about sex, gender (identity/presentation), and sexual orientation and race, ethnicity and racism.

Reporting on sex and gender	*Use the terms sex (biological attribute) and gender (shaped by social and cultural circumstances) carefully in order to avoid confusing both terms. Indicate if findings apply to only one sex or gender; describe whether sex and gender were considered in study design; whether sex and/or gender was determined based on self-reporting or assigned and methods used. Provide in the source data disaggregated sex and gender data, where this information has been collected, and if consent has been obtained for sharing of individual-level data; provide overall numbers in this Reporting Summary. Please state if this information has not been collected. Report sex- and gender-based analyses where performed, justify reasons for lack of sex- and gender-based analysis.*
Reporting on race, ethnicity, or other socially relevant groupings	*Please specify the socially constructed or socially relevant categorization variable(s) used in your manuscript and explain why they were used. Please note that such variables should not be used as proxies for other socially constructed/relevant variables (for example, race or ethnicity should not be used as a proxy for socioeconomic status). Provide clear definitions of the relevant terms used, how they were provided (by the participants/respondents, the researchers, or third parties), and the method(s) used to classify people into the different categories (e.g. self-report, census or administrative data, social media data, etc.) Please provide details about how you controlled for confounding variables in your analyses.*
Population characteristics	*Describe the covariate-relevant population characteristics of the human research participants (e.g. age, genotypic information, past and current diagnosis and treatment categories). If you filled out the behavioural & social sciences study design questions and have nothing to add here, write "See above."*
Recruitment	*Describe how participants were recruited. Outline any potential self-selection bias or other biases that may be present and how these are likely to impact results.*
Ethics oversight	*Identify the organization(s) that approved the study protocol.*

Note that full information on the approval of the study protocol must also be provided in the manuscript.

Field-specific reporting

Please select the one below that is the best fit for your research. If you are not sure, read the appropriate sections before making your selection.

☐ Life sciences ☐ Behavioural & social sciences ☒ Ecological, evolutionary & environmental sciences

For a reference copy of the document with all sections, see nature.com/documents/nr-reporting-summary-flat.pdf

Ecological, evolutionary & environmental sciences study design

All studies must disclose on these points even when the disclosure is negative.

Study description	The study describes changes in observed niche characteristics of microbial assemblages during marine heatwaves. Niche characteristics were defined by molecular census and based on relative abundance measures along oceanographic gradients.
Research sample	Microbial (Bacterial, Archaeal, Eukaryote) assemblages in 2 Litres seawater, in accordance with the protocols of the Australian Microbiome (https://confluence.csiro.au/display/ASM/Ausmicrobiome+Scientific+Manual)
Sampling strategy	Samples were collected either monthly during Integrated Marine Observing System (IMOS) National Reference Station (NRS) Cruises

Sampling strategy	or during oceanographic research voyages on an opportunistic basis. Depth rangefrom surface to 6000m, temperatures range from -2 to 32C and latitude ranges from 0-66S.
Data collection	Samples were collected by various groups. All data were generated by the Australian Microbiome (https://www.australianmicrobiome.com/) in accordance with its published protocols (https://confluence.csiro.au/display/ASM/Ausmicrobiome+Scientific+Manual)
Timing and spatial scale	IMOS sampling is ongoing with data here described until Jun2021. Monthly temporal sampling began in 2012 at 3 IMOS NRS (Mariai Island, Port Hacking and North Stradbroke Island) in June 2012 and a further two (Rottnest Island, and Yongala) in June 2015. Quarterly sampling begain in June 2015 at two further IMOS NRS (Darwin, Kangaroo Island). Samples collected on Southern Hemisphere oceanographic voyages were carried out on an opportunistic basis between 2010 and 2019 and span latitudes 0-66 S.
Data exclusions	No data were excluded
Reproducibility	No experiments were carried out
Randomization	Samples were allocated into groups based on whether they were collected during a marine heatwave event (MHW) or during non-marine heatwave conditions (non-MHW). Heatwave events were defined as periods of at least five consecutive days when daily sea-surface temperatures (SSTs) exceed the 90th percentile of climatological (seasonal) SST observations (calculated between 1/1/1982 and 31/12/2011)
Blinding	Blinding not relevant

Did the study involve field work? ☒ Yes ☐ No

Field work, collection and transport

Field conditions	All oceanographic conditions coincident with sampling are reported and available alongside molecular census data at the Australian Microbiome. All IMOS NRS data is available on the Australian Ocean Data Network portal (https://portal.aodn.org.au/) and all oceanographuc cruise data is available on the Commonwealth Scientific and Industrial Research Organisation (CSIRO) Data Trawler (https://www.cmar.csiro.au/data/trawler/)
Location	Samples were collected across various Southern Hemisphere ocean ecosystems. Information for all IMOS NRS stations are cited and available at Brown et al 2018 Scientific Data 5, Article number: 180130. All information concerning the locations accessed by cruises of the R/V Southern Surveyor (ss2010_v09, ss2012_t07, ss2013_t03), the R/V Aurora Australis (AA2014/15_v2, AA2015/16_v3) and the R/V Investigator (IN2014_e03, IN2015_c02, IN2015_v03, IN2016_t02, IN2016_v03, IN2016_v04, IN2017_v01, IN2019_v03) are available via the Australian Marine National Facility (https://mnf.csiro.au/en/About/About-us) and are also available with sample
Access & import/export	IMOS and MNF are Australian Government facilities and all samples are collected in line with national and international requirements including appropriate permits
Disturbance	no disturbance was caused by this study

Reporting for specific materials, systems and methods

We require information from authors about some types of materials, experimental systems and methods used in many studies. Here, indicate whether each material, system or method listed is relevant to your study. If you are not sure if a list item applies to your research, read the appropriate section before selecting a response.

Materials & experimental systems

n/a	Involved in the study	
☒	☐	Antibodies
☒	☐	Eukaryotic cell lines
☒	☐	Palaeontology and archaeology
☐	☒	Animals and other organisms
☒	☐	Clinical data
☒	☐	Dual use research of concern
☐	☒	Plants

Methods

n/a	Involved in the study	
☒	☐	ChIP-seq
☒	☐	Flow cytometry
☒	☐	MRI-based neuroimaging

Animals and other research organisms

Policy information about studies involving animals; ARRIVE guidelines recommended for reporting animal research, and Sex and Gender in Research

Laboratory animals	The study did not involve laboratory animals
Wild animals	Microorganisms were collected by filtration of 2Litres of natural seawater through a 0.22um filter.
Reporting on sex	Organisms are asexual
Field-collected samples	All samples collected from the field were stored at -80C prior to analysis.
Ethics oversight	No ethics approval or guidance was required as the target of the study was microorgansims

Note that full information on the approval of the study protocol must also be provided in the manuscript.

Dual use research of concern

Policy information about dual use research of concern

Hazards

Could the accidental, deliberate or reckless misuse of agents or technologies generated in the work, or the application of information presented in the manuscript, pose a threat to:

No	Yes	
☒	☐	Public health
☒	☐	National security
☒	☐	Crops and/or livestock
☒	☐	Ecosystems
☒	☐	Any other significant area

Experiments of concern

Does the work involve any of these experiments of concern:

No	Yes	
☒	☐	Demonstrate how to render a vaccine ineffective
☒	☐	Confer resistance to therapeutically useful antibiotics or antiviral agents
☒	☐	Enhance the virulence of a pathogen or render a nonpathogen virulent
☒	☐	Increase transmissibility of a pathogen
☒	☐	Alter the host range of a pathogen
☒	☐	Enable evasion of diagnostic/detection modalities
☒	☐	Enable the weaponization of a biological agent or toxin
☒	☐	Any other potentially harmful combination of experiments and agents
